# Characterizing and mapping the spatial variability of HIV risk among adolescent girls and young women: A cross-county analysis of population-based surveys in Eswatini, Haiti, and Mozambique

**DOI:** 10.1371/journal.pone.0261520

**Published:** 2021-12-17

**Authors:** Kristen N. Brugh, Quinn Lewis, Cameron Haddad, Jon Kumaresan, Timothy Essam, Michelle S. Li

**Affiliations:** 1 Palladium, Washington, DC, United States of America; 2 Fraym, Arlington, VA, United States of America; 3 Office of HIV/AIDS, United States Agency for International Development, Washington, DC, United States of America; University of Salamanca, SPAIN

## Abstract

**Background:**

To stem the HIV epidemic among adolescent girls and young women (AGYW), prevention programs must target services towards those most at risk for HIV. This paper investigates approaches to estimate HIV risk and map the spatial heterogeneity of at-risk populations in three countries: Eswatini, Haiti and Mozambique.

**Methods:**

We analyzed HIV biomarker and risk factor data from recent population-based household surveys. We characterized risk using three approaches: complementary log-log regression, latent class analysis (LCA), and presence of at least one risk factor. We calculated the proportion and 95 percent confidence intervals of HIV-negative AGYW at risk across the three methods and employed Chi-square tests to investigate associations between risk classification and HIV status. Using geolocated survey data at enumeration clusters and high-resolution satellite imagery, we applied algorithms to predict the number and proportion of at-risk AGYW at hyperlocal levels.

**Results:**

The any-risk approach yielded the highest proportion of at-risk and HIV-negative AGYW across five-year age bands: 26%-49% in Eswatini, 52%-67% in Haiti, and 32%-84% in Mozambique. Using LCA, between 8%-16% of AGYW in Eswatini, 37%-62% in Haiti, and 56%-80% in Mozambique belonged to a high vulnerability profile. In Haiti and Mozambique, the regression-based profile yielded the lowest estimate of at-risk AGYW. In general, AGYW characterized as “at risk” across the three methods had significantly higher odds of HIV infection. Hyperlocal maps indicated high levels of spatial heterogeneity in HIV risk prevalence and population density of at-risk AGYW within countries.

**Conclusion:**

Characterizing risk among AGYW can help HIV prevention programs better understand the differential effect of multiple risk factors, facilitate early identification of high-risk AGYW, and design tailored interventions. Hyperlocal mapping of these at-risk populations can help program planners target prevention interventions to geographic areas with populations at greatest risk for HIV to achieve maximal impact on HIV incidence reduction.

## Background

Adolescent girls and young women (AGYW) aged 10–24 are at disproportionate risk of HIV. AGYW accounted for a quarter of new infections in sub-Saharan Africa in 2019, more than twice their 10% share of the total population. Among adolescents ages 15–19, girls accounted for five in six new HIV infections and were 4.5 times more likely to be infected than boys [1, 2]. Young women ages 15–24 are approximately three times more likely to be infected by HIV in both the Caribbean and in Eastern and Southern Africa [2].

Although risk and vulnerability factors for HIV infection among AGYW are well-established in the scientific literature [3], there is increasing acknowledgment that vulnerability to HIV is multidimensional; risk factors are not consistently associated with transmission across different contexts; and risks are unevenly distributed, particularly across age groups and locations. HIV prevention programs note the need to identify and engage the AGYW who are most vulnerable to HIV acquisition. This includes better characterization of risk factors that are most positively correlated with HIV acquisition [4–9] and improved understanding of the differential effects of multiple risk factors in the derivation of vulnerability profiles and risk typologies [10, 11].

In addition to understanding how risk factors contribute to HIV vulnerability among AGYW, HIV prevention programs also need to understand variation in risk typologies at local levels. Recent studies have examined the spatial concentration of vulnerability and HIV infection to advocate risk-based geographic microtargeting of prevention services to specific populations and locations, with a focus on key populations and other high-risk groups [12–17]. Studies by Nutor and colleagues in Malawi and Mozambique used multivariate logistic regression to model predictors of adult positive HIV status and then applied spatial interpolation at the cluster level to map HIV prevalence stratified by significant risk factors [18, 19]. Cuadros, et al., analyzed Demographic and Health Survey (DHS)-geotagged HIV serological biomarker data from 20 countries in sub-Saharan Africa and found that geographic variability in HIV risk factors drive HIV prevalence heterogeneities among adults [20]. Research focused on youth populations has also found that geospatial clustering of HIV is associated with the clustering of risk behavior and vulnerability factors in high-endemic countries [21–23] and that risk and vulnerability factors do not entirely overlap geographically [24].

The DREAMS (Determined, Resilient, Empowered, AIDS-free, Mentored, and Safe) Partnership of the U.S. President’s Emergency Plan for AIDS Relief (PEPFAR) aims to address the underlying economic, educational, and structural factors that make AGYW vulnerable to HIV infection by providing a comprehensive, multisectoral package of core interventions to alleviate them. This strategy requires identifying geographic areas where the need is greatest, estimating the number of AGYW in need of prevention services, and targeting interventions aimed at addressing high-impact risk factors in this population [25]. Local data on subnational variation in HIV risk among AGYW are critical if we are to target and allocate limited resources and interventions more efficiently and reduce the number of new HIV infections. To support DREAMS program planning, we analyzed (1) the combination of factors that contribute to HIV risk among AGYW, and (2) the spatial heterogeneity of risk among AGYW ages 10–24 in three high-burden countries (Eswatini, Haiti, and Mozambique). We also considered risk factors for women ages 25–29 in Eswatini, based on age eligibility for the DREAMS program in the country. Estimated HIV prevalence among young women ages 15–24 varies across these countries (1.0% in Haiti, 6.5% in Mozambique, and 13.0% in Eswatini in 2019), however, in all three countries women are between 2 to 3 times more likely to be living with HIV compared to their male peers [2]. These three countries were selected based on presence of the DREAMS program, geographical variation, and availability of recent publicly-accessible data for analysis.

## Methods

### Data sources

We conducted a secondary analysis of publicly available nationally representative, cross-sectional household surveys containing individual-level HIV outcome and risk factor data: the 2017 Haiti Demographic and Health Survey (HDHS) [26], the 2015 Mozambique Standard AIDS Indicator Survey (MAIS) [27], and the 2016–2017 Swaziland HIV Incidence Measurement Survey 2 (SHIMS2) [28]. All three surveys provide representative estimates of HIV prevalence at the national and first-level administrative division (region in Eswatini, department in Haiti, province in Mozambique) for AGYW who are 15–24 years old; 2016–2017 SHIMS2 HIV prevalence data are also nationally representative for AGYW 10–14 years.

The detailed methodology of Demographic and Health Surveys and Population-based HIV Impact Assessments have been previously described [29–31]. Briefly, these surveys use a two-stage stratified cluster sampling design to randomly select enumeration areas (EA) and households. All women above age 15 in Eswatini and between 15 to 49 in Haiti and Mozambique in the sampled households were selected to respond to standard household and women’s questionnaires. In Haiti, two-thirds of selected households also responded to questions on child discipline for one child during the month before the survey. In Eswatini, one adult woman in each household was randomly selected to answer questions on her experiences with violence, and participants aged 10–14 responded to the young adolescent questionnaire. All women in Eswatini, and women aged 15 to 49 in two out of three selected households in Haiti and Mozambique were eligible for HIV biomarker testing. All children (0–14 years) in 50% of the household sub-sample were eligible for blood draw and HIV testing. Additional details on survey sampling methods, data collection procedures and ethical considerations can be found in the final survey reports [26–28].

### Variables

#### HIV risk and vulnerability factors

We used PEPFAR’s 2020 Country Operational Plan (COP20) Guidance [25] to inform our selection of independent variables for analysis as risk factors. We considered dichotomous risk factors for self-reported sexual behaviors (such as multiple sexual partners, sexually transmitted infection (STI), no or inconsistent condom use, and transactional sex), experiences of violence, substance use, and self or caregiver reported information on school enrollment and orphanhood. There were slight variations in the definitions of variables and in data availability based on survey questionnaire design, sampling, methods across countries, and country program context on eligibility for the DREAMS program. A detailed description of variable construction is described in S1 Table. The study countries tracked very few risk factors for the 10–14 age band. Table 1 summarizes the availability of data by survey and age band on the risk factors we analyzed in this study.

**Table 1:** Study variables by survey and age band.

Variable	Haiti 2017 DHS			Mozambique 2015 AIS			Eswatini 2017 SHISM2			
	10–14	15–19	20–24	10–14	15–19	20–24	10–14	15–19	20–24	25–29
HIV status derived from biomarker testing		✓	✓		✓	✓	✓	✓	✓	✓
**Sexual behaviors**										
Self-reported STI status (past 12 months)		✓	✓		✓	✓				
Sexually active (past 4 weeks)		✓	✓		✓	✓				
Inconsistent condom use (past 12 months)		✓	✓		✓	✓		✓	✓	✓
No condom use at last sex (past 12 months)								✓	✓	✓
Early sexual debut^a^		✓	✓		✓	✓		✓	✓	✓
Transactional sex (past 12 months)^b^		✓	✓					✓	✓	✓
Multiple sex partners (past 12 months)		✓	✓		✓	✓		✓	✓	✓
Age disparate sex (past 12 months)^c^		✓	✓		✓	✓		✓	✓	✓
**Experiences of violence** ^**d**^										
Ever forced sex		✓	✓		✓	✓				
Ever experienced physical violence		✓	✓		✓	✓				
Ever experienced violence (physical, sexual, or emotional)^e^		✓	✓		✓	✓	✓			
Current experience of violence in past 12 months (physical and/or sexual)^e^								✓	✓	✓
Current experience of violence in past month (physical discipline)	✓									
Exposure to childhood physical abuse in household (past month)	✓									
**Substance use (at time of survey)**										
Consumes alcohol regularly		✓	✓							
Alcohol use^f^								✓	✓	✓
Uses tobacco		✓	✓							
**Orphanhood** ^**g**^										
Single orphan	✓	✓		✓	✓					
Double orphan	✓	✓		✓	✓					
Orphanhood (either single or double)							✓	✓		
**Schooling** ^**h**^										
Not currently enrolled in school	✓	✓					✓	✓		
Never attended or not currently enrolled in school				✓	✓	✓				

^a^Early sexual debut is defined as having had first sexual intercourse at or before age 15.

^b^Transactional sex is defined as having had sex, or been sexually involved with someone, in exchange for cash, gifts, or other material support in the 12 months preceding the survey.

^c^Age disparate sex is defined as having any reported sexual partner more than 10 years older, out of one’s last three sexual partners.

^d^Questions pertaining to violence were only asked of AGYW 17+ in Mozambique.

^e^Reference periods for experience of violence variables differ by age band in Eswatini: the reference period is ever experienced violence for AGYW under age 15 years, and past 12 months for AGYW 15+.

^f^Regular alcohol consumption (Haiti) defined as drinks alcohol every day or every now and then; alcohol misuse (Eswatini) defined as ever having used alcohol for AGYW 10–14 years per the 2017 SHIMS2 adolescent survey module and as consuming alcohol two or more times per week for AGYW 15+ per the adult survey module.

^g^Questions on orphan status were asked on a limited basis among people ages 18 years and older across all study countries.

^h^Questions related to school enrollment were asked of all respondents, however the use of this variable for AGYW 18+ in Haiti and Eswatini may be less relevant as a risk factor as does not distinguish between AGYW who have completed their education and AGYW who have dropped out or never attended school.

COP20 guidelines state that vulnerability and risk criteria considered for DREAMS enrollment should be relevant to specific age bands. Although orphanhood and being out of school are considered vulnerabilities for younger AGYW, school enrollment is less common among older AGYW. Based on country program context, we excluded current school enrollment for AGYW 18+ years in Haiti and Eswatini, and in Mozambique we included AGYW in that age group who either were not currently enrolled in school or had never attended school.

#### Outcome

We considered positive HIV status as the primary outcome variable in the construction and validation of risk profiles. The 2017 HDHS and the 2015 MAIS conducted random biomarker testing among adults ages 15–49 years (Haiti) and 15–59 years (Mozambique) in two-thirds of sampled households; AGYW 10–14 years were not eligible for HIV testing. The 2016–2017 SHIMS2 conducted biomarker testing of all rostered and consenting adults ages 15 and older and offered biomarker testing to all children ages 0–14 years in half of sampled households. Among our population of interest, there were high coverage rates for HIV testing as part of the study surveys (ranging from 87.8% in Mozambique to 98.6% in Haiti).

### Data access and preparation

The HDHS, MAIS, and SHIMS2 data are publicly available and can be accessed electronically after approval from the DHS and Population-Based HIV Impact Assessment programs. These survey instruments employ age restrictions and skip patterns for questions about sexual history. They do not ask respondents ages 10–14 years in any country about sexual activity. For AGYW ages 15+ years, we constructed analytical variables to include AGYW who had sexually debuted and engaged in the risky behavior as “yes” and recoded all other AGYW as “no” if they had never had sex or if they had sexually debuted but did not engage in the risky behavior.

The final analytical sample for age groups with HIV testing results included only respondents who had conclusive HIV blood test results and were assigned non-zero HIV testing weights in the survey data sets. To generate risk profiles for AGYW who were at risk and HIV-negative, we took those AGYW who had been classified as at-risk and HIV-positive and reclassified them as not at risk across all models and countries. Because HIV testing was not conducted for AGYW ages 10–14 in the HDHS and MAIS, our analytic samples for this age band in Haiti and Mozambique included all AGYW who had responded to questions about risk factors. Though this approach may have captured some girls who are HIV-positive, research has found that HIV prevalence in adolescents ages 10–14 years is generally low compared to that of 15- to 19-year-olds and similar to rates found in young children [32]. A set of flow charts for the derivation of analytical samples in each country is depicted in S1–S3 Figs.

### Statistical analyses

To understand the relevance of various risk factors in AGYW 15+ years in the study samples, we assessed the relationships between HIV status and the independent variables using chi-square tests. We then compared three methodological approaches to develop HIV vulnerability typologies for the identification of at-risk AGYW. First, we considered AGYW to be at risk if they exhibited any risk factor listed in Table 1. The analytical sample for this approach included all AGYW with data for at least one non-missing risk factor. Among age groups for which HIV testing was conducted, a conclusive HIV test result and assignment of a non-zero biomarker survey weight were also required. Second, we developed complementary log-log (clog-log) binary regression models in each country to analyze factors associated with positive HIV status. Regression models accounted for stratification and clustering in the sample design. Models were run using a pooled 15–24 age band in Haiti and Mozambique, and a pooled 15–29 age band in Eswatini; AGYW 10–14 years were not included in this approach, because of the lack of HIV outcome data and limited risk factor data. To retain maximum sample size, our analysis considered only independent variables with the highest response rates (greater than 80%). We did not consider additional control variables in the regression models in order to align as closely as possible with COP20 risk factors. To estimate an empirically optimal cut point for the predicted probability of an adolescent girl or young woman acquiring HIV, Youden’s J statistic [33] was calculated, using the “cutpt” command in Stata [34]. We classified AGYW with predicted probabilities of HIV infection higher than this cut point as “at risk”. We reported area under the receiver operating characteristics (AUROC) curve statistics as performance measures for the estimated cut points. Third, we used latent class analysis (LCA) to identify groups of AGYW with similar risk characteristics [35]. A series of LCA models with two to three classes that accounted for complex survey design and clustering in each country were examined. We used the criteria of minimizing the values for Akaike Information Criterion (AIC) and Bayesian Information Criterion (BIC), combined with practical and theoretical usefulness of the final class structure to determine model fit [36, 37]. All risk factors listed in Table 1 were used in the LCA, with positive HIV status as the outcome of primary interest. The analytic sample for the LCA models across all countries included all AGYW 15+ years with conclusive HIV blood test results, non-zero blood test sample weights, and at least one non-missing risk factor. We created a dichotomous risk class by combining in a single “at-risk” group latent classes indicative of high risk and vulnerability exposures, and similarly combining in a single “not at-risk” group latent classes indicative of low risk and vulnerability exposures.

For each risk model, Pearson’s Chi-square tests were employed as a robustness check to assess associations between risk classification and HIV status. All statistical analyses were conducted using Stata, Version 16. We report unweighted sample sizes and weighted, cluster-adjusted point estimates with 95% confidence intervals (CIs). Biomarker sample weights were applied for all AGYW in Eswatini and for AGYW 15–24 years in Haiti and Mozambique. Individual sample weights were applied for AGYW 10–14 years in Haiti and Mozambique [31, 38].

### Spatial interpolation of risk at hyperlocal levels

To understand subnational variation in risk typologies, we produced spatial maps of the distribution of HIV acquisition risk at lower geographic levels. To determine the spatial variations of HIV risk at the cluster level, we analyzed geomasked location coordinates for the centroid of each survey enumeration area from the 2017 HDHS and the 2015 MAIS. As the 2016–2017 SHIMS2 did not include publicly available georeferenced data, we did not produce spatial maps of the distribution of HIV risk at the sub-national level for Eswatini.

Spatial layers were created from the HDHS and MAIS individual-level risk profiles via artificial intelligence (AI)/machine learning (ML) algorithms that predict the proportion of at-risk and HIV-negative AGYW at a resolution of one square kilometer. This strategy builds on tested methods for interpolation of spatial data [39]. The AI/ML algorithm yields a spatial ensemble model that identifies correlations between the survey data at enumeration clusters and high-resolution satellite imagery and related derived data products, including earth observation data and gridded population information (i.e., human settlement mapping and biophysical surfaces). The model can be used to predict the survey data for all non-enumerated areas. The DHS program pioneered a similar approach in 2015 [40]. We performed a series of quality checks on the spatial layers produced, including the comparison of the spatial layer’s output to the survey at its level of representativeness (national and/or first-level administrative division), and confirmed that the spatial layer’s output aligned with the survey. Conducting all spatial analysis in R(version 3.6.2), we then combined these low-geographic-level risk estimates with estimates of population density from the WorldPop database [41] to generate population estimates for AGYW at risk. ArcGIS Pro (version 2.6.0) was used to construct production-quality maps.

**Fig 1:**
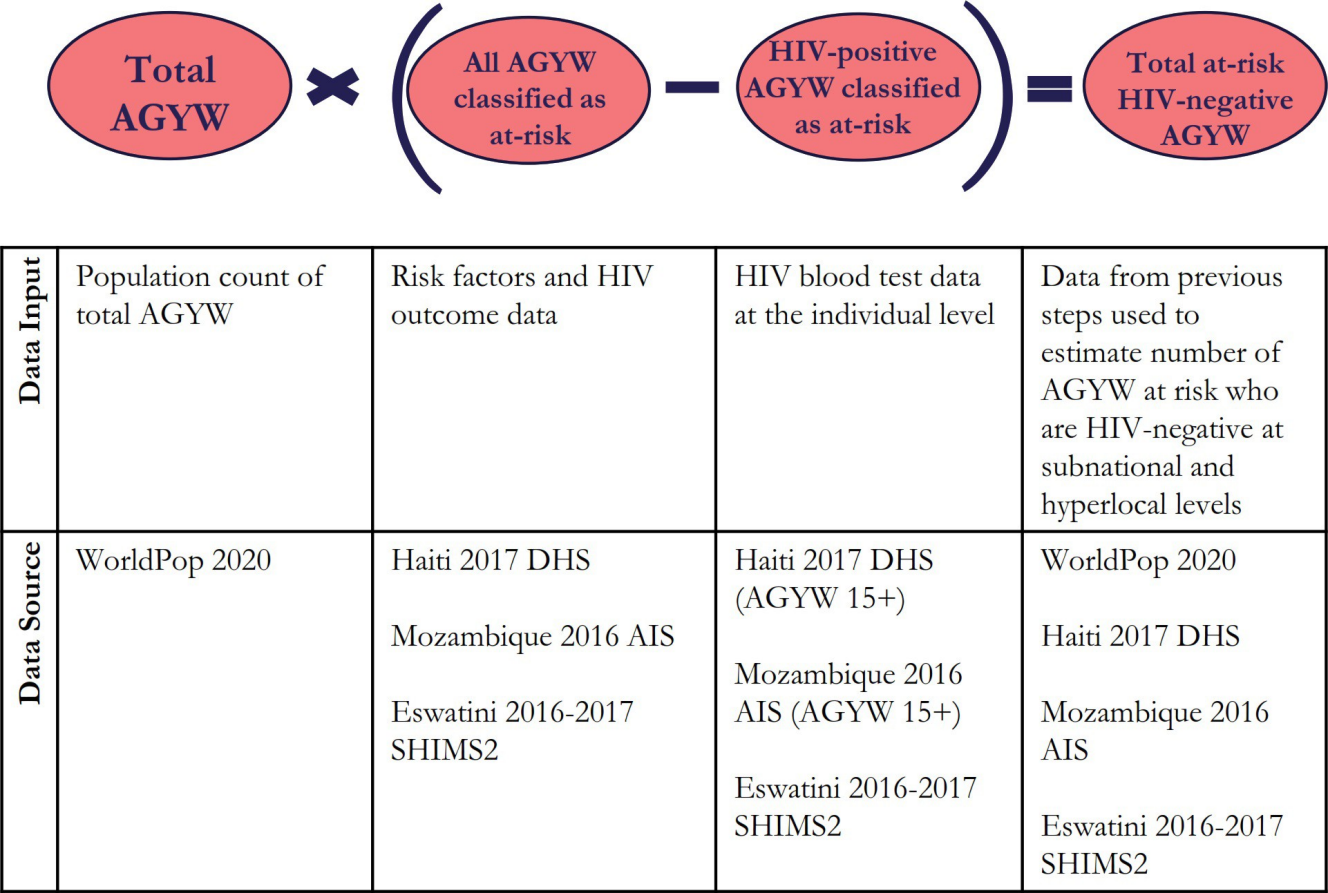
Population size estimation process: AGYW who are HIV-negative and at risk for HIV infection.

Fig 1 shows the estimation and mapping processes, data inputs, and data sources that we used. There is some country-specific variation in at-risk models and the spatial interpolation process depending upon the availability of risk factor survey data and access to geotagged survey data.

## Results

### Characteristics of the study samples

Descriptive characteristics of the study samples by HIV status for each country and age band are presented in S2 Table. In general, HIV prevalence among AGYW was lowest in Haiti and highest in Eswatini and increased by age band; HIV prevalence rates varied widely among countries and age bands.

We considered risk factors related to violence, orphanhood, and schooling among AGYW ages 10–14 years. Approximately 13% of AGYW 10–14 years in Haiti, 14% in Mozambique, and 18% in Eswatini had lost a parent. While more than two-thirds of AGYW 10–14 years reported personal experiences of physical violent discipline in the past month in Haiti, less than 10% in Eswatini reported ever experiencing physical violence. In Mozambique, the most common risk factor among adolescent girls was not being currently enrolled in or never attending school.

Risk and vulnerability factors considered for AGYW 15+ years included sexual behaviors, experiences of violence, substance use, and schooling. Approximately one-quarter of AGYW in Haiti and one-third in Mozambique had their first sexual intercourse at the age of 15 or younger; early sexual debut was significantly associated with HIV status in all groups with sufficient sample size. Across the countries, between 8 to 16 percent of AGYW ages 15–19 reported inconsistent condom use and between 13 to 20 percent of AGYW ages 20–24 reported age-disparate sex. Lifetime prevalence of physical violence was high among AGYW 15+ years in Haiti and Mozambique, and significantly associated with HIV positivity in the 20–24 age band. About 90% of AGYW 15–19 years in Haiti were currently enrolled in school, compared to 63% in Mozambique who either were not currently enrolled or had never attended school. Current school enrollment among 15- to 19-year-olds in Eswatini differed significantly by HIV status.

### Any-risk profiles

We categorized AGYW as “at any risk” if they exhibited any risk factor. The proportion of these AGYW by country is shown in Table 2. Roughly similar proportions of AGYW were categorized as at any risk in Haiti across age bands, but there were large increases in the proportion of AGYW categorized as at any risk between the 10–14 and 15–19 age bands in Eswatini and Mozambique.

**Table 2:** Percentage of AGYW who are classified as at-risk of HIV infection, by estimation methodology.

	Any-Risk Profile			Regression-Based Profile			LCA-Based Profile		
	N	%^a^	95% CI ^a^	N	% ^a^	95% CI ^a^	N	% ^a^	95% CI ^a^
**Haiti**									
10–14	3249	58.94	57.01, 60.80	-			-		
15–19	2231	52.85	50.15, 55.53	2092	22.91	20.87, 25.85	2231	37.04	34.61, 39.54
20–24	1812	69.26	66.41, 71.97	1652	37.41	34.61, 40.29	1812	63.28	60.25, 66.21
**Mozambique**									
10–14	2518	32.18	29.33, 35.17	-			-		
15–19	1371	80.97	78.47, 83.24	1198	55.47	51.55, 59.32	1371	60.84	57.45, 64.12
20–24	1222	97.31	96.28, 98.07	975	64.07	59.53, 68.37	1222	91.92	90.06, 93.46
**Eswatini**									
10–14	578	28.22	23.96, 32.47	-			-		
15–19	1031	40.30	37.34, 43.26	983	10.92	8.58, 13.26	1031	10.22	8.06, 12.37
20–24	895	62.37	58.96, 65.78	801	30.69	27.17, 34.21	895	22.52	19.52, 25.53
25–29	811	72.50	68.98, 76.01	744	32.07	28.80, 35.33	811	21.61	18.92, 24.30

^a^Weighted by sampling probability weights.

Note: Sample denominators (N) include both HIV-positive and HIV-negative AGYW. Numerators include HIV-positive and HIV-negative AGYW who are classified as at-risk. For AGYW 10–14 year in Haiti and Mozambique, the denominator (N) includes all girls with at least one non-missing risk factor and the numerator is restricted to AGYW classified as at-risk.

Table 3 summarizes the percentage of AGYW who are HIV-positive, by risk class (S3 Table provides contingency tables of HIV status and risk classification for all study models). In general, HIV positivity in the at-risk group is significantly higher than in the not-at-risk group for most study models.

**Table 3:** Prevalent HIV infection by risk profile methodology.

Profile	Age	N	At-risk		Not at-risk		
			% ^a^	95% CI ^a^	% ^a^	95% CI ^a^	p-value^a^
**Haiti**							
Any-risk	15–19	2231	0.65	0.30, 1.41	0.00	0.00, 0.00	0.018
	20–24	1812	2.52	1.71, 3.72	0.70	0.22, 2.17	0.027
Regression	15–19	2092	0.43	0.13, 1.43	0.35	0.13, 0.91	0.798
	20–24	1652	3.96	2.49, 6.24	0.94	0.49, 1.80	< 0.001
LCA	15–19	2231	0.70	0.28, 1.75	0.13	0.03, 0.54	0.031
	20–24	1812	2.71	1.83, 4.00	0.68	0.25, 1.86	0.008
**Mozambique**							
Any-risk	15–19	1371	7.72	5.83, 10.15	1.73	0.41, 6.99	0.024
	20–24	1222	13.26	11.12, 15.77	4.23	0.94, 17.08	0.095
Regression	15–19	1198	7.86	5.49, 11.13	4.09	2.55, 6.51	0.028
	20–24	975	15.82	12.88, 19.30	6.59	4.35, 9.86	< 0.001
LCA	15–19	1371	8.54	6.19, 11.66	3.54	2.10, 5.90	0.003
	20–24	1222	13.23	10.96, 15.89	10.70	6.043, 18.25	0.483
**Eswatini**							
Any-risk	10–14	578	6.97	3.74, 12.63	2.06	1.04, 4.05	0.005
	15–19	1031	11.97	9.04, 15.70	3.90	2.47, 6.18	< 0.001
	20–24	895	21.78	18.15, 25.92	19.42	15.16, 24.54	0.430
	25–29	811	34.82	30.53, 39.37	44.43	36.77, 52.37	0.028
Regression	15–19	983	17.38	11.76, 24.92	6.01	4.41, 8.16	< 0.001
	20–24	801	31.26	25.61, 37.52	15.75	12.60, 19.52	< 0.001
	25–29	744	47.74	40.01, 55.05	31.16	26.33, 36.43	< 0.001
LCA	15–19	1031	16.83	11.00, 24.87	6.07	4.49, 8.16	< 0.001
	20–24	895	26.89	21.00, 33.73	19.15	16.06, 22.68	0.023
	25–29	811	43.80	35.23, 52.75	35.72	31.62, 40.04	0.080

^a^Weighted by sampling probability weights.

^a^p-values are reported for Chi-square tests of difference in HIV positivity by risk category.

### Regression-based risk profiles

Table 4 presents the results from the clog-log regression models. Early sexual debut was significantly associated with HIV status in all countries, and age-disparate sex was significantly associated with positive HIV status in Mozambique and Eswatini. Predicted probabilities were calculated for all AGYW in the regression estimation samples and AGYW were classified as at risk if their predicted probability was greater than the empirically optimal cut point. The three models had similar AUROC values at the optimal cut points (0.65 in Haiti, 0.60 in Mozambique, and 0.62 in Eswatini), indicating modest discriminating ability for predicting HIV status.

**Table 4:** Complimentary log-log regression results.

	Haiti, 15–24 years (N = 3744)		Mozambique, 15–24 years (N = 2173)		Eswatini, 15–29 years (N = 2528)	
Risk factor	ME	SE	ME	SE	ME	SE
History of STI in the past 12 months	0.00616	0.0063	0.0469*	0.0248	N/A	--
Sexually active in the past 4 weeks	0.00174	0.0044	-0.0132	0.0161	N/A	--
Early sexual debut	0.0115**	0.0049	0.0354**	0.0163	0.133***	0.0223
Multiple sex partners in the past 12 months	-0.00536	0.0125	0.0501*	0.0279	0.045	0.0354
Age disparate sex	0.00635	0.0057	0.0643***	0.0155	0.1080***	0.0202
Inconsistent condom use in last 12 months	Omitted	--	Omitted	--	0.00767	0.0171
Transactional sex	Omitted	--	N/A	--	0.0292	0.0469
Alcohol misuse	0.00839*	0.0051	N/A	--	0.1460	0.0956
Uses tobacco	0.00022	0.0132	N/A	--	N/A	--
Never attended or not currently enrolled in school	Omitted	--	0.0321	0.0196	N/A	--
**Empirical optimal cut point estimation (Youden method)**						
Empirical optimal cut point	0.0124		0.0668		0.1840	
AUROC curve at cut point	0.65		0.60		0.62	
**HIV prevalence**	1.1%		9.4%		21.2%	

ME = marginal effect, SE = standard error, AUROC = area under the receiving operating characteristics.

N/A: Data on risk factor was not collected in the country survey.

Risk factors were omitted from regression models due to missing values, insufficient response rates, and lack of variation among HIV-positive and HIV-negative AGYW.

*p<0.1,

**p<0.05,

***p<0.01.

AGYW were classified as at risk of their predicted probability of HIV-positivity was greater than the empirical optimal cut points. Table 2 summarizes the proportion of AGYW classified as at risk under the regression-based risk profile. Although fewer AGYW were classified as at risk using the regression-based approach compared to the at-any-risk approach, similar patterns in risk can be seen across countries. Across age bands, Mozambique had the higher proportion of AGYW at risk, followed by Haiti, then Eswatini.

AGYW classified as at risk in the regression analysis were significantly more likely to be HIV-positive compared to those classified as not at risk in all age bands in Mozambique and Eswatini. However, HIV prevalence did not differ by regression-based risk classification among AGYW 15–19 years in Haiti.

### LCA-based risk profiles

We compared AIC and BIC model fit indices for two- and three-class models to determine the optimal number of latent classes, variables, analytical samples, and best fit models (LCA GOF statistics appear in S4 Table). Within each country-specific risk model, the three-class solutions produced consistently lower AIC and BIC values than the two-class models did. Table 5 presents the conditional response probabilities (latent class marginal means) for each risk factor, marginal probabilities of class membership, and HIV prevalence by the latent classes for each country. The expected class for each individual adolescent girl or young woman in the LCA analytical sample was determined based on the maximum posterior probability assignment rule, whereby individuals are assigned to their class of highest predicted probability. The percentages of AGYW classified in each latent group approximated the conditional probabilities of class membership estimated from model parameters.

**Table 5:** LCA conditional response probabilities and membership classification.

	Haiti (N = 4043)			Mozambique (N = 2593)			Eswatini (N = 2737)		
	Response Probability			Response Probability			Response Probability		
**Risk and vulnerability factor**	**Class 1**	**Class 2**	**Class 3**	**Class 1**	**Class 2**	**Class 3**	**Class 1**	**Class 2**	**Class 3**
Have any STI (past 12 months)	0.019	0.111	0.166	0.010	**1.000**	0.000	Omitted		
Sexually active (past 4 weeks)	0.044	0.399	**0.686**	0.160	**0.680**	**0.610**	Omitted		
Early sexual debut	0.038	0.376	**0.528**	0.120	0.290	**0.430**	0.050	0.270	0.180
Multiple sex partners (past 12 months)	0.000	0.036	0.086	0.000	0.190	0.040	0.010	0.110	0.200
Age disparate sex	0.000	0.113	0.205	0.000	0.210	0.190	0.060	0.220	0.290
Inconsistent condom use (past 12 months)	0.022	0.238	**0.557**	0.000	**0.690**	**0.460**	0.290	**0.600**	**0.850**
Transactional sex	0.000	0.029	0.063	Omitted			0.000	0.100	0.080
Ever forced sex/history of sexual violence	0.000	0.274	0.000	Omitted			0.000	0.000	0.000
Ever experienced physical violence/history of physical violence	0.000	**0.886**	0.000	Omitted			0.000	**1.000**	0.000
Ever experienced any violence/history of any violence	0.000	**1.000**	0.014	Omitted			0.000	**1.000**	0.000
Alcohol misuse^a^	0.036	0.089	0.111	Omitted			0.000	0.020	0.020
Uses tobacco	0.005	0.039	0.030	Omitted			Omitted		
Single orphan	0.180	0.285	0.108	Omitted			0.280	**0.640**	0.270
Double orphan	0.020	0.033	0.054	Omitted			0.080	0.000	0.150
Not currently enrolled in school/never attended^b^	0.165	**0.504**	**0.650**	**0.440**	**0.710**	**0.890**	0.000	**1.000**	**0.730**
Orphan status / experience of violence (sexual and/or physical)^c^	N/A			0.240	**0.920**	**0.530**	N/A		
Marginal probability of latent class membership	0.478	0.305	0.217	0.304	0.102	0.593	0.714	0.029	0.257
HIV prevalence within class (%)	0.3	2.1	1.5	4.7	20.1	10.7	18.9	46.3	26.7
Classification of AGYW (%)	51.8	29.2	19	27.9	4.8	67.3	82.3	1.6	16
Classification of AGYW (n)	2096	1179	768	723	126	1744	2256	44	437

Bold values denote the key distinguishing characteristic of the latent class profile.

^a^Defined as consumes alcohol regularly (Haiti) and alcohol misuse (Eswatini).

^b^Defined as not currently enrolled in school (Haiti and Eswatini) and not currently enrolled or never attended school (Mozambique).

^c^Orphan status and experience of violence were combined into a single risk factor for AGYW ages 15–24 in Mozambique as questions on orphan status were only asked of respondents under the age of 18 and questions on experiences of violence were only asked of respondents aged 18+.

We used a cut point of 0.4 to define a response probability as high. Across the countries, Class 1 had the lowest probabilities of each risk factor and the lowest HIV prevalence among the three latent classes. In Haiti, the Class 2 profile had the highest probabilities of individuals reporting experiences of sexual, physical, or any violence; this class had the highest HIV prevalence. The Class 3 profile reported the highest probabilities of sexual behaviors. In Mozambique, both Class 2 and Class 3 profiles had high probabilities of recent sexual activity, inconsistent condom use, poor school attendance, and orphan status or experience of violence. The Class 2 profile in Mozambique also reported a 100% likelihood of STI. In Eswatini, Class 2 and Class 3 profiles had high probabilities of inconsistent condom use, and not currently enrolled in school; the main distinguishing characteristic of Class 2 was the 100% likelihood of experiencing physical or any violence.

Across all countries, Classes 2 and 3 were combined in a single at-risk category to estimate the percentage of AGYW classified as at risk (Table 2). Similar patterns in risk can be seen across countries: Mozambique had the highest proportion of girls classified as at risk across all age bands, followed by Haiti and Eswatini. In Eswatini, the LCA-based approach yielded the lowest estimates of proportion of AGYW at risk across the methods.

AGYW classified as at risk based on LCA risk profiles were significantly more likely to be HIV-positive than those AGYW classified as not at risk across all age bands in Haiti and Eswatini. However, there was no statistical difference in HIV prevalence by risk classification among AGYW 20–24 years in Mozambique (Table 3).

### Variation in at-risk and HIV-negative estimates

To generate the target population of prevention efforts, HIV-positive AGYW initially classified as at risk (Table 2) were reclassified as not at risk to generate risk prevalence, at-risk population size estimates, and at-risk heat maps of AGYW who were both HIV-negative and classified as at risk. We were unable to differentiate risk classification by HIV status for AGYW 10–14 years in Haiti and Mozambique owing to the lack of HIV status data in the 2017 HDHS and 2015 MAIS. Table 6 presents the percentages of AGYW who were HIV-negative and classified as at risk by the risk profile method. We compared 95% CIs to understand whether at-risk estimates differed significantly. However, conclusions regarding lack of statistical differences between risk estimates when CIs did overlap should be made with caution, because this approach was overly conservative.

**Table 6:** Percentage of AGYW who are classified as at risk and HIV-negative, by method.

	Any-Risk Profile			Regression-Based Profile			LCA-Based Profile		
	N	%	95% CI	N	%	95% CI	N	%	95% CI
**Haiti**									
10–14	3249	58.94	57.01, 60.80	---			---		
15–19	2231	52.51	49.78, 55.22	2092	22.81	20.77, 24.99	2231	36.78	34.34, 39.29
20–24	1812	67.51	64.73, 70.17	1652	35.93	33.21, 38.73	1812	61.56	58.62, 64.42
**Mozambique**									
10–14	2518	32.18	29.33, 35.17	---			---		
15–19	1371	74.72	71.80, 77.43	1198	51.11	47.10, 55.11	1371	55.64	52.08, 59.15
20–24	1222	84.40	81.75, 86.72	975	53.93	49.45, 58.35	1222	79.75	76.59, 82.60
**Eswatini**									
10–14	578	26.25	22.05, 30.45	---			---		
15–19	1031	35.47	32.70, 38.24	983	9.02	7.02, 11.03	1031	8.50	6.70, 10.29
20–24	895	48.78	45.09, 52.47	801	21.10	17.93, 24.27	895	16.47	13.82, 19.11
25–29	811	47.25	43.35, 51.16	744	16.85	13.93, 19.76	811	12.14	9.69, 14.60

Note: Sample denominators (N) include both HIV-positive and HIV-negative AGYW. Numerators are restricted to those AGYW who are both classified as at risk and HIV-negative. For AGYW 10–14 years in Haiti and Mozambique the denominator (N) is all girls with at least one non-missing risk factor, and the numerator is restricted to AGYW classified as at-risk.

In Haiti, there was no overlap of the 95% CIs within each profile method and within each age band across profile methods, indicating these estimates were significantly different from one another. In Mozambique, the 95% CIs for regression-based risk profile estimates overlapped across age bands and profile methods, suggesting that the estimates were not significantly different. Among 15- to 19-year-old AGYW in Eswatini, CIs overlapped for regression-based and LCA-based models; these multivariate risk profiles produced risk estimates that were one-fourth the magnitude of those risk estimates generated by the categorical any-risk profiles. Results from Eswatini suggested that the percentage of AGYW who were HIV-negative and classified as at risk was similar across the 20–24 and 25–29 age bands in each model.

#### Variation in risk estimates among subnational units

**Fig 2:**
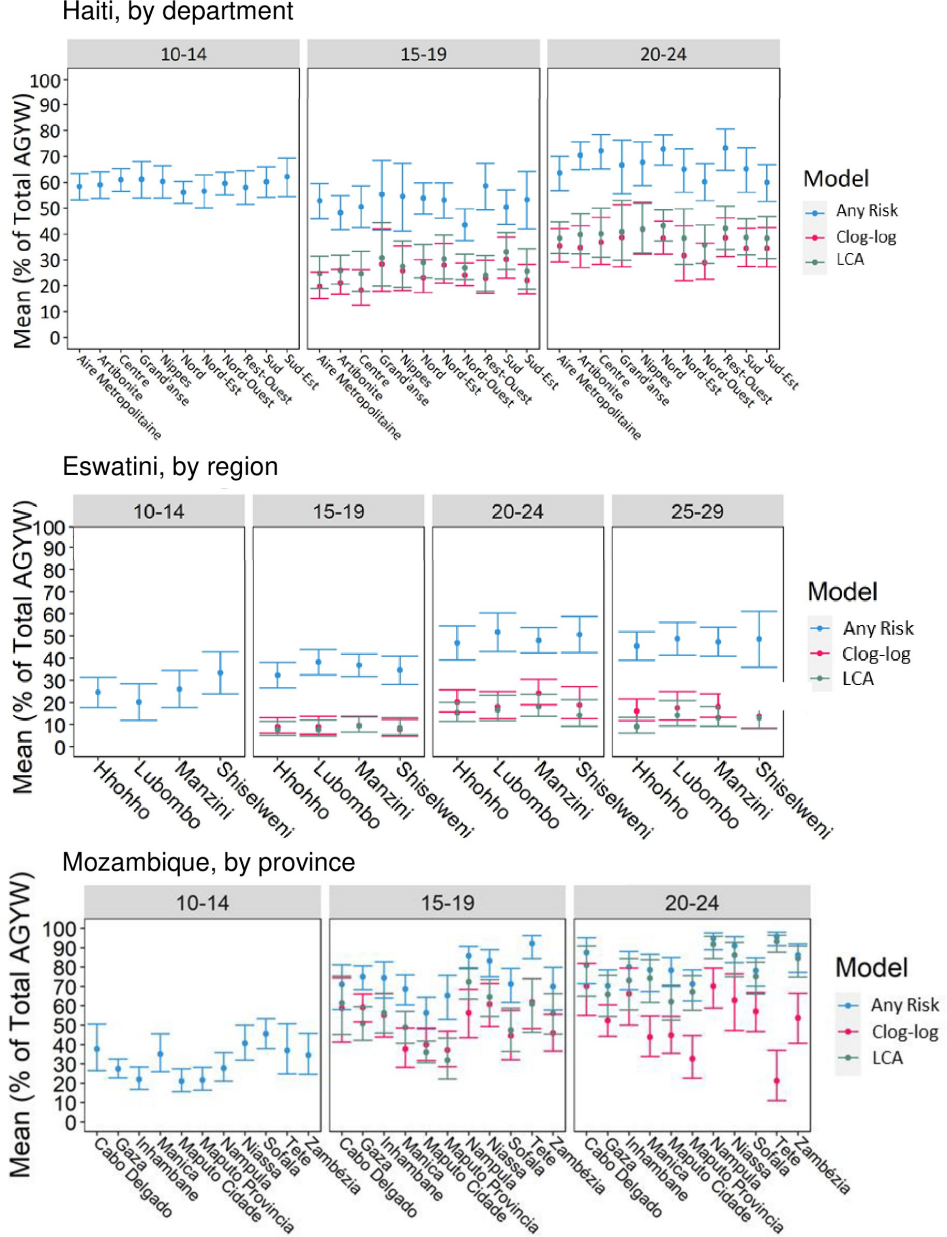
Subnational comparison of risk prevalence estimates, by method (mean with 95% CI). Percentage of AGYW who are HIV-negative and classified as at-risk of HIV infection by country, age band, subnational unit, and model. (A) Haiti–Department level. (B) Mozambique–Provincial level. (C) Eswatini–Regional level.

Fig 2 presents the mean and 95% CIs of AGYW classified as at risk and HIV-negative at the subnational level for each study country, risk profile method, and age band. We did not find evidence of overlap between the any-risk estimates and the regression-/LCA-based estimates for AGYW 15+ years in any department in Haiti or region in Eswatini, but there was considerable overlap among the three models in Mozambique. In general, age-band/method-specific risk estimates were stable at the subnational levels in Haiti (Fig 2A) and Eswatini (Fig 2C). Conversely, there was substantial subnational variation in estimates and 95% CIs within Mozambique (particularly in the clog-log estimates); CIs did not overlap across several provinces for each risk model within each age band.

The precision of subnational estimates varied across administrative units, estimation methods, and age bands. For example, CIs were wider for all risk estimation methods among AGYW 15–19 years in Grand’Anse and Nippes, in Haiti, suggesting higher variation and/or less precision among these departments. In Tete province, in Mozambique, the any-risk CIs were tightest for AGYW 15+ years, the clog-log CIs were the widest among AGYW 15+ years, and LCA-based CIs were wide among 15–19-year-olds but narrow among 20- to 24-year-olds.

### Hyperlocal heat maps of AGYW at risk for HIV infection

**Fig 3:**
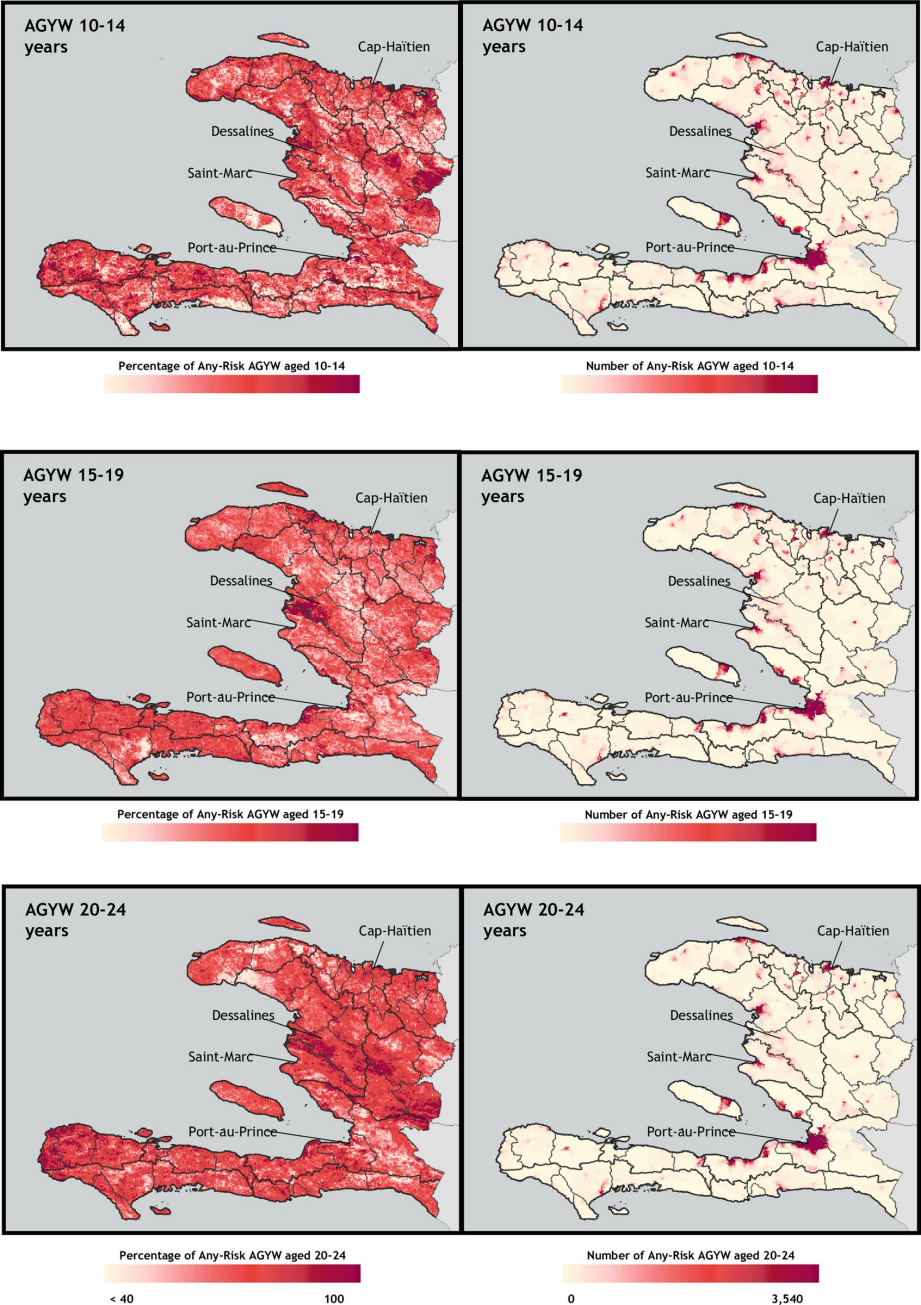
Hyperlocal heat maps of HIV risk prevalence (percentage at risk) and population density (number at risk), based on any-risk profile in Haiti, by age band. The number-at-risk is calculated as those AGYW who are both classified as at-risk and HIV-negative for AGYW ages 15+ and the number-at-risk for AGYW 10–14 years is calculated as those AGYW classified as at-risk. Base map and data from the Spatial Data Repository, The Demographic and Health Surveys Program.

**Fig 4:**
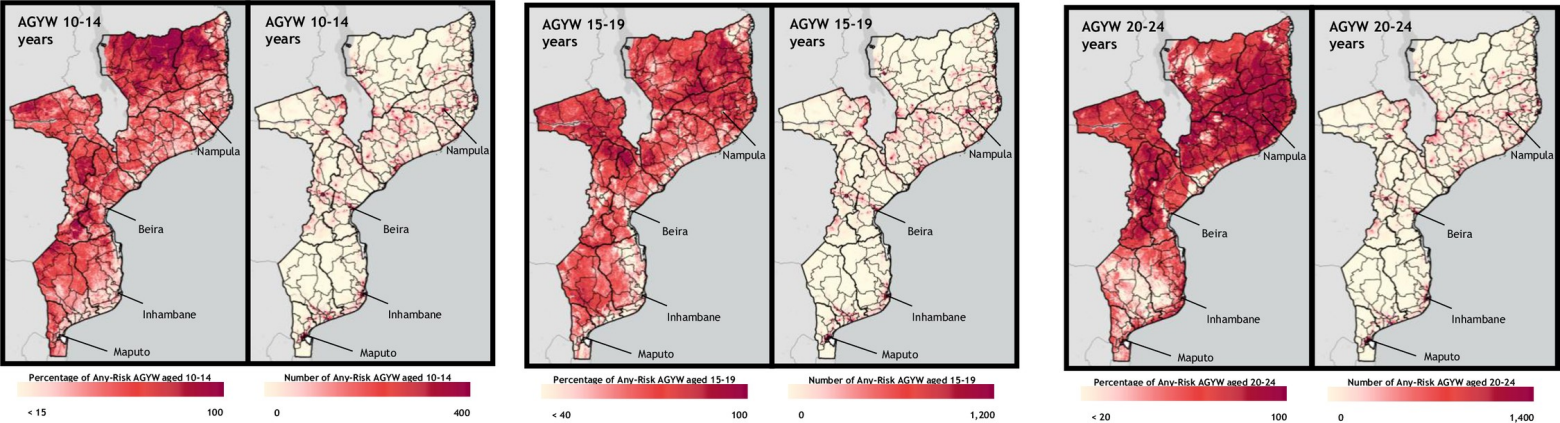
Hyperlocal heat maps of HIV risk prevalence (percentage at risk) and population density (number at risk), based on any-risk profile in Mozambique, by age band. The number-at-risk is calculated as those AGYW who are both classified as at-risk and HIV-negative for AGYW ages 15+, and the number-at-risk for AGYW 10–14 years is calculated as those AGYW classified as at-risk. Base map and data from the Spatial Data Repository, The Demographic and Health Surveys Program.

We selected the “at any risk” method to develop hyperlocal heat maps of HIV risk. Figs 3 and 4 shows the geospatial distribution of the percentage and number of AGYW who were HIV-negative and at risk of infection, by age band, for Haiti (Fig 3) and Mozambique (Fig 4). Despite low district- and regional-level variation in at-risk prevalence (within the same age band and model), as illustrated in Fig 2, each country map shows substantial heterogeneity in the risk prevalence and population density of AGYW at risk for HIV infection. While a greater number of at-risk AGYW were identified in areas with a greater number of AGYW overall, risk prevalence maps do not necessarily mirror population density.

In Haiti, although the greatest number of at-risk AGYW lived in Port-Au-Prince across all age bands, risk prevalence was much higher in and around Dessalines and Cap Haïtien. Visualizing spatial heterogeneity of risk prevalence also highlighted areas of high risk prevalence outside these main urban centers, which was not as evident in the population-based maps. Among 20- to 24-year-olds, the western-most departments of Haiti—particularly Anse-d’Hainault arrondissement—had very high risk prevalence rates.

Mozambique followed patterns similar to those in Haiti. The greatest number of at-risk AGYW lived in major urban areas, such as Nampula, Beira, Inhambane, and Maputo. Risk prevalence in these areas was relatively low among 10- to 14-year-olds and 15- to 19-year-olds, whereas risk prevalence among 20- to 24-year-olds was very high everywhere, including these urban areas. Although the maps showing the number of AGYW at risk showed similar patterns across all age bands, the risk prevalence maps were markedly different. Among 20- to 24-year-olds, risk prevalence tended to be greatest in areas with higher population—in northern and central Mozambique and along the coast. Among 10- to 14-year-olds and 15- to 19-year-olds, the reverse was true: risk prevalence was lower in urban areas and in the more populated northern provinces where population was higher, risk prevalence was lower.

## Discussion

This study used the most recent and comprehensive publicly available geolocation data on risk factors and HIV prevalence to develop distinct profiles of HIV vulnerability among AGYW and to understand intranational spatial risk heterogeneity. It makes several contributions to the knowledge base. We included adolescent girls ages 10–14 years in our analyses, and to our knowledge this is the first study to compare risk profile methods and to develop maps for program targeting based on risk rather than HIV incidence or prevalence. Our results advance knowledge of using risk profiles as appropriate to identify at-risk AGYW, because HIV prevalence was nearly always significantly higher among AGYW in at-risk groups. This study also adds important information on spatial variation in HIV risk: Heat maps show that AGYW at-risk population density and at-risk prevalence do not always overlap geographically, and program planners can leverage this information to assess trade-offs between allocating prevention resources to areas of highest risk prevalence compared to areas with the largest number of at-risk AGYW. This can be especially important in rural areas which may be overlooked by HIV prevention programs due to low population density, despite risk prevalence that may be just as high or higher than in major urban centers. Triangulation of HIV at-risk population density with other HIV prevention program indicators such as mother-to-child transmission rates, coverage of voluntary medical male circumcision rates, and viral suppression rates can further inform the geographical prioritization and effective resource allocation of HIV prevention programming, especially for oral pre-exposure prophylaxis (PrEP) targeting.

Recent studies of risk factors for HIV among AGYW 15–24 years have identified multiple sexual partners, age-disparate sex, STI symptoms, and alcohol use as significant risk factors for new HIV infection or HIV prevalence [4, 5, 7]. An important advancement of our research is the exploration of risk factors separately by age band and country. Compared to these previous studies, the present study does not find significant associations among STI symptoms and alcohol use with HIV status across study countries. Age-disparate sex is not an important characteristic of at-risk AGYW ages 15+ in Haiti but is highly significant in regression-based risk profiles in Mozambique and Eswatini. Early sexual debut is the only risk factor that we found to be significantly associated with HIV risk across all age bands and study countries in the regression analyses. We also found that AGYW in LCA class with the highest HIV prevalence also had high probabilities of experiencing violence across all study countries. Findings from this analysis can be translated to develop or adapt risk assessment and screening tools to better identify AGYW at high risk of HIV acquisition for eligibility into HIV prevention programs.

An additional advancement made by this study is the estimation of HIV risk prevalence among AGYW. Much of the previous literature stops at the identification of risk and vulnerability correlates of HIV infection, without describing the distribution of risk among study populations. Recent research employing LCA derives proportion of populations at risk based on estimated vulnerability profiles. Comins, et al., analyzed patterns of structural and social determinants of HIV/STI acquisition among AGYW 15–24 years old in Ethiopia. They assigned 17% to the highly vulnerable group characterized by higher probabilities of early sexual debut, transactional sex, age-disparate sex, and experiences of physical or sexual violence, and had an HIV/STI prevalence rate twice as high as the low vulnerability groups [10]. Mathur and colleagues used LCA to identify HIV vulnerability classes among out-of-school AGYW ages 15–24 years in Kenya, Zambia, and Malawi. Although they found that the high vulnerability profile had significantly higher odds of HIV-related risks than did the low-vulnerability profile, there was not always a significant difference in HIV prevalence between vulnerability groups across countries [11]. Our research extends these approaches by estimating risk prevalence by AGYW age band in three study countries using three different vulnerability classification methods. Similar to LCA study results in Ethiopia and Kenya, we found that HIV prevalence is nearly always higher among AGYW in at-risk age groups than in other AGYW.

In addition, we estimated the percentage of AGYW who were both at risk and HIV-negative. This proportion is salient for identifying and estimating the size of target populations in need of HIV prevention services. For example, in a program with the scale of the PEPFAR-supported DREAMS initiative, decision makers might use this information to identify gaps in the coverage of HIV prevention programs and to inform resource allocation decisions related to program expansion or scale down. The use of AGYW at-risk burden estimates has already been used to inform DREAMS target setting and program expansion decisions [42].

In this study, we found that the any-risk profile produces the highest estimates of the percentage of AGYW who both are at risk and HIV-negative across age bands (among AGYW 15+ years) and countries. The any-risk profiles result in broad at-risk estimates because they include AGYW who may have only a single risk or vulnerability factor, even if that factor is not significantly associated with HIV status. This inclusive approach ensures that girls are not overlooked in HIV prevention programming but may overestimate the percentage of AGYW at risk and result in less efficient targeting and use of prevention resources.

Conversely, the regression-based risk estimation approach is the least inclusive. This method may miss key areas of HIV transmission, particularly when HIV prevalence and risk prevalence do not align. The LCA approach serves as a middle ground. Although this approach does not necessarily sort AGYW into classes based on risk factors that are significantly associated with HIV infection, the models it yields provide a more nuanced understanding of risk. In our models, the regression-based risk profiles provided the lowest estimates of risk prevalence across all age bands 15+ years in Haiti and Mozambique. However, the LCA-based risk profile had the lowest percentage of at-risk AGYW age 15+ years in Eswatini, potentially due to the relatively low percentage of AGYW across age bands that exhibited multiple risky sexual behaviors and lack of variation in risk factors exhibited. It will be important to understand the focus, needs and phase (start up, scale up, maintenance) of HIV prevention programs to determine the most appropriate risk estimation approaches. For example, a more inclusive approach may be appropriate for comprehensive, HIV prevention education programs whereas a narrower approach focusing on those at greatest risk of HIV acquisition may be appropriate for ARV-based prevention products like PrEP.

As expected, our results confirm that not all AGYW are at equal risk; risk estimates vary across age bands, methods, and across and within countries. We found variation in risk across higher-level administrative units in study countries, and the any-risk profile maps show high-resolution variation in AGYW at risk and HIV-negative population distributions and risk prevalence.

An interesting finding of this study is that HIV risk prevalence among HIV-negative AGYW does not track with HIV prevalence in a consistent way. In a lower prevalence setting like Haiti, between 22 to 68 percent of AGYW are estimated to be at risk and HIV-negative based on results across models. In Mozambique, a country with high HIV prevalence, risk prevalence is higher than 50 percent across all models for AGYW age 15+ years. In another high HIV prevalence country like Eswatini, risk prevalence is not universally high overall or in comparison with age-specific HIV prevalence. Specifically, the LCA-based risk profiles produce risk prevalence estimates that are lower than HIV prevalence among 20- to 24-year-olds and 25- to 29-year-olds. These findings align with results from previous studies, which concluded that variability in sexual risk behavior does not necessarily follow HIV prevalence heterogeneities, [20] and that risk and vulnerability factors do not entirely overlap [24]. These results, taken together with variations in at-risk AGYW population and at-risk prevalence geospatial distributions, reinforce the importance of considering multiple contextual factors when using at-risk profiles to inform prevention population and geographic programming targets.

Our study has some limitations. This analysis relies on cross-sectional data, which limits our ability to infer causality or temporality between risk factors and HIV positivity. Survey data are self-reported retrospectively AGYW and may be subject to recall bias and/or underreporting. A major limitation in using HIV prevalence data among adolescents is that a proportion of the girls and young women ages 10–19 years living with HIV may have acquired HIV through mother-to-child-transmission [3]. Due to small sample sizes and the lack of HIV outcome data for children under age 15 in Haiti and Mozambique, we could apply only the any-risk profile approach to this age group. Further, comparisons of results across countries may be to be interpret with caution due to variations in survey modules, questionnaire design, HIV testing methods, and variable construction across the DHS and PHIA surveys.

We made several assumptions in our analyses owing to data availability and access restrictions. Because we could not identify HIV-positive and HIV-negative AGYW 10–14 years in Haiti and Mozambique, the final estimate of the percentage of AGYW who are at risk and HIV-negative included some HIV-positive girls. We assumed that HIV prevalence is low among this age band, which is consistent with lower HIV prevalence in the 15–19 years age band. We used this same approach in Eswatini for consistency among methods. Similarly, we did not have subnational estimates of the number of AGYW who are HIV-positive. As a result, we had to incorporate HIV-positive girls in our final size estimations by making the percentage of AGYW at risk out of all AGYW. Combining the percentage of AGYW at risk with subnational estimates of HIV prevalence (e.g., Naomi estimates [43]) could improve the accuracy of HIV risk estimates among HIV-negative AGYW. We also assumed that the proportion of AGYW at risk and HIV-negative remained constant between the time of survey data collection (2017 for Haiti, 2015 for Mozambique, and 2016–2017 for Eswatini) and when we paired these data with 2020 WorldPop population projections.

## Conclusion

Evidence both of risk and spatial heterogeneity in HIV infection requires program planners to consider two epidemic typologies simultaneously when they target and prioritize prevention efforts: risk/vulnerability and geographic/spatial. This study investigated three methods for developing HIV acquisition risk profiles among AGYW using publicly available data and risk factors germane to prominent bilateral prevention programs. We also demonstrated an application of AI/ML spatial interpolation to produce hyperlocal heat maps of AGYW at risk and HIV-negative population densities and risk prevalence to support comprehensive program planning and efficient prevention resource targeting. These methods could be considered in similar contexts with high-quality geospatial, HIV outcome, and HIV risk factor data among AGYW. Future work should assess the feasibility and practical use of these approaches in micro- and macro-targeting of at-risk AGYW for HIV prevention programs.

## Supporting information

S1 Fig Analytical sample derivation flowcharts for Haiti 2017 DHS. There were 3249 AGYW ages 10–14 years with at least one non-missing risk factor. AGYW age 15+ were eligible for HIV testing in 2/3 of selected households.
(PDF)

S2 Fig Analytical sample derivation flowcharts for Mozambique 2015 AIS. There were 2518 AGYW ages 10–14 years with at least one non-missing risk factor. AGYW age 15+ were eligible for HIV testing in 2/3 of selected households.
(PDF)

S3 Fig Analytical sample derivation flowcharts for Eswatini 2017 SHIMS2. Biomarker testing was offered to all rostered and consenting adults (15+ years) in all sampled households and to all children 0–14 years in 50% of sampled households.
(PDF)

S1 Table Definitions of risk factor variables used in the risk estimation approaches across Eswatini, Haiti and Mozambique. (DOCX)

S2 Table Descriptive statistics by HIV status. (DOCX)

S3 Table Unweighted contingency tables. (DOCX)

S4 Table LCA goodness-of-fit model statistics. (DOCX)
