# Supplementary material for: Characterizing and mapping the spatial variability of HIV risk among adolescent girls and young women: A cross-county analysis of population-based surveys in Eswatini, Haiti, and Mozambique
Source: PLoS One. 2021 Dec 17;16(12):e0261520. doi: 10.1371/journal.pone.0261520 (PMC8682891; doi:10.1371/journal.pone.0261520)

S2 Fig: Analytical sample derivation flowchart for Mozambique 2015 AIS

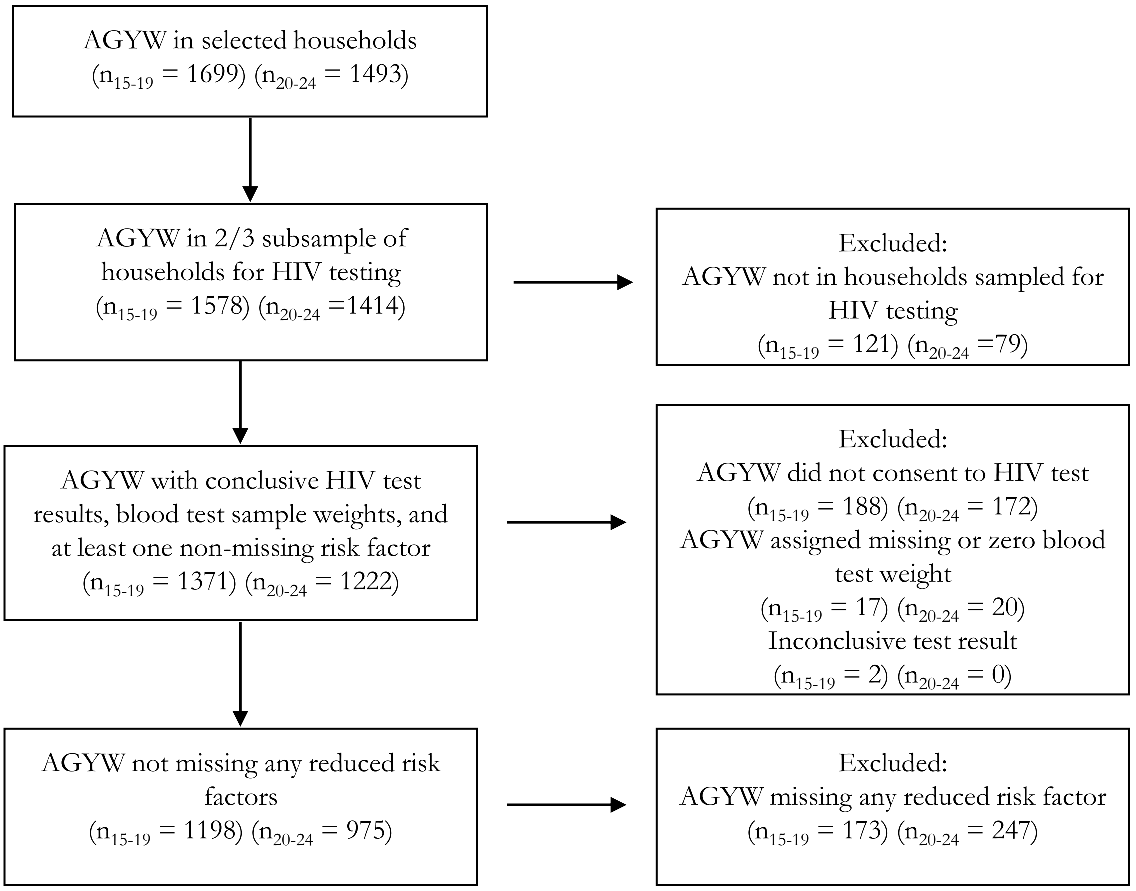

Notes: There were 2518 AGYW age 10-14 years in Mozambique with at least one non-missing risk factor. AGYW age 15+ were eligible for HIV testing in 2/3 of selected households. No AGYW with conclusive HIV test results and non-zero, non-missing blood test survey weights were missing all risk factors.

S2 Fig: Analytical sample derivation flowchart for Mozambique 2015 AIS

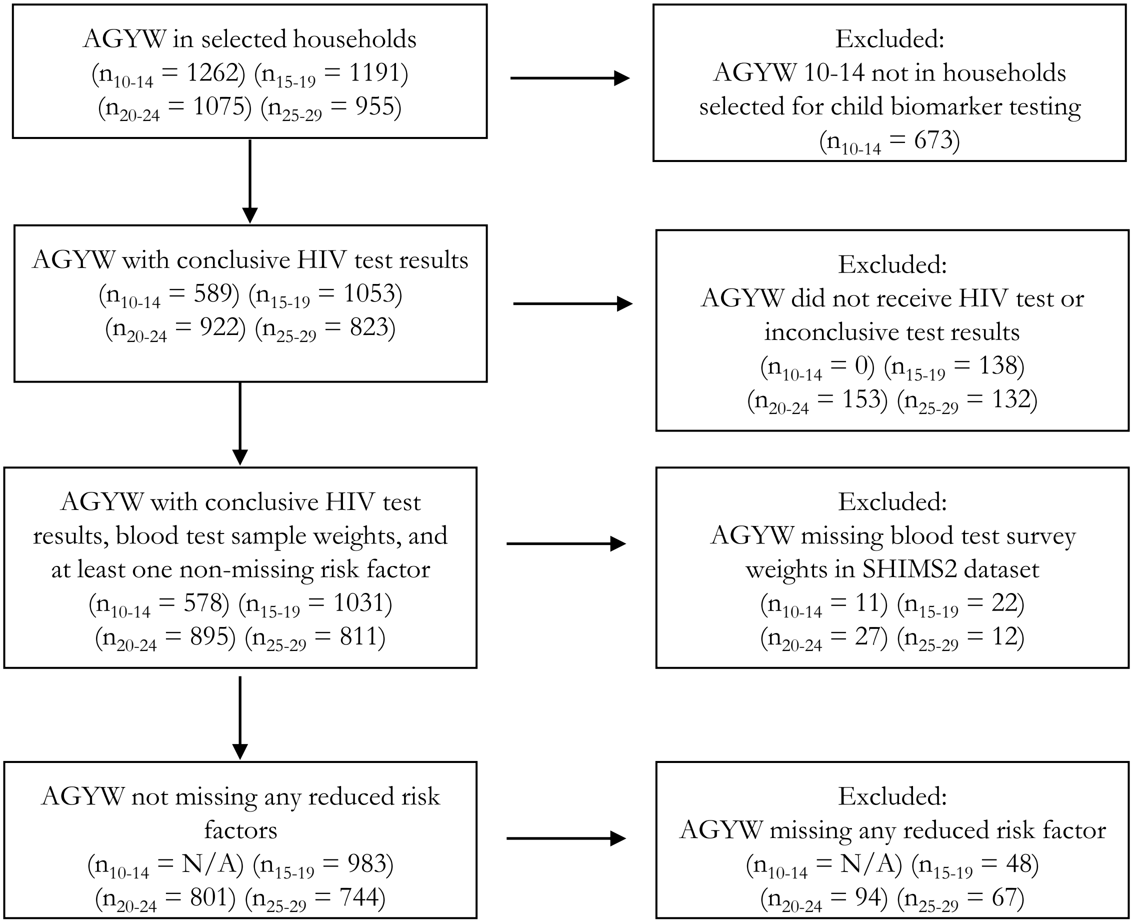

Supplement: S2 Fig — There were 2518 AGYW ages 10–14 years with at least one non-missing risk factor. AGYW age 15+ were eligible for HIV testing in 2/3 of selected households. (PDF) [file pone.0261520.s002.pdf]
