# Supplementary material for: Characterizing and mapping the spatial variability of HIV risk among adolescent girls and young women: A cross-county analysis of population-based surveys in Eswatini, Haiti, and Mozambique
Source: PLoS One. 2021 Dec 17;16(12):e0261520. doi: 10.1371/journal.pone.0261520 (PMC8682891; doi:10.1371/journal.pone.0261520)
Supplement: S3 Fig — Biomarker testing was offered to all rostered and consenting adults (15+ years) in all sampled households and to all children 0–14 years in 50% of sampled households. (PDF) [file pone.0261520.s003.pdf]

S3 Fig: Analytical sample derivation flowchart for Eswatini 2016-2017 SHIMS2

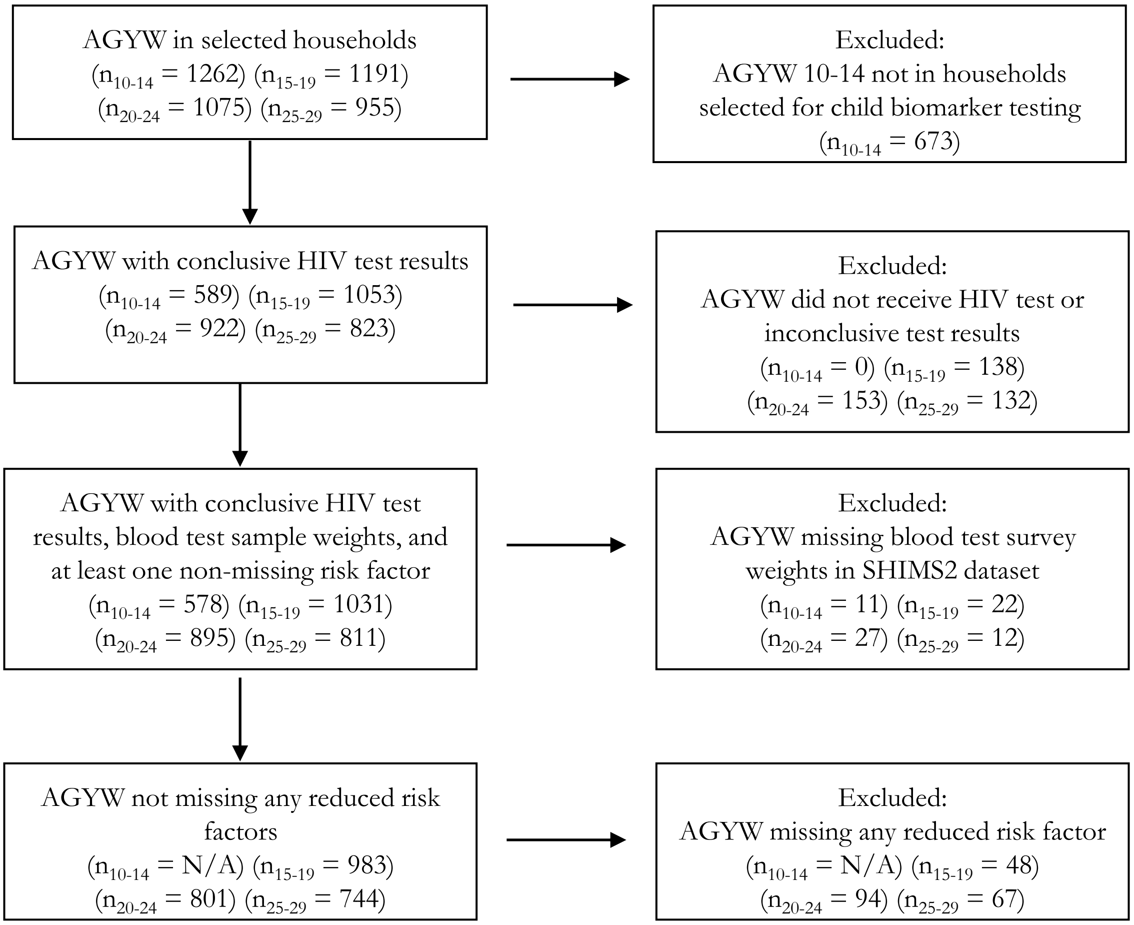

Notes: Biomarker testing was offered to all rostered and consenting adults (15+ years) in all sampled households and to all children 0-14 years in 50% of sampled households in Eswatini. No AGYW with conclusive HIV test results and non-zero, non-missing blood test survey weights were missing all risk factors.
