# Supplementary material for: Characterizing and mapping the spatial variability of HIV risk among adolescent girls and young women: A cross-county analysis of population-based surveys in Eswatini, Haiti, and Mozambique
Source: PLoS One. 2021 Dec 17;16(12):e0261520. doi: 10.1371/journal.pone.0261520 (PMC8682891; doi:10.1371/journal.pone.0261520)
Supplement: S1 Table — (DOCX) [file pone.0261520.s004.docx]

S1 Table. Definitions of risk factor variables used in the risk estimation approaches across Eswatini, Haiti and Mozambique

Definitions of risk factor variables differed across countries, depending on data availability, survey questionnaire design, sampling methods across countries, and input from country program representatives on construction of indicators and cut-offs.

| Risk Factor | **Measurement and Binary Variable Definition** | | |
| --- | --- | --- | --- |
|  | **Eswatini** | **Haiti** | **Mozambique** |
| Self-reported STI status (past 12 months) | Not included. | Defined as reporting having had genital sore/ulcer or genital discharge in the last 12 months | Defined as reporting having had genital sore/ulcer or genital discharge in the last 12 months |
| Sexually active (past 4 weeks) | Not included. | Coded as ‘active in the last 4 weeks’ based on the question ‘*When was the last time you had sexual intercourse?*’ | Coded as ‘active in the last 4 weeks’ based on the question ‘*When was the last time you had sexual intercourse?*’ |
| Inconsistent condom use (past 12 months) | Defined as reporting ‘most of the time’, ‘sometimes’, ‘rarely’ or ‘never’ with at least one of up to the last three partners the respondent had sex with in the last 12 months, in response to the question “*In the last 12 months, how often did you use condoms with [this partner] when having vaginal or when having anal sex?”*  or  Reporting ‘no’ in response to the question “*the last time you had sex with [partner], was a condom used?”* for at least one of the last three sexual partners in the last 12 months | Defined as reporting ‘no’ with at least one of the last three partners the respondent had sex with in the last 12 months, in response to the question “*Was a condom used every time you had sexual intercourse with this person in the last 12 months*?” | Defined as reporting ‘no’ with at least one of the last three partners the respondent had sex with in the last 12 months, in response to the question “*Was a condom used every time you had sexual intercourse with this person in the last 12 months*?” |
| No condom use at last sex (past 12 months) | Defined as reporting ‘no’ in response to the question “*the last time you had sex with [partner], was a condom used?”* for at least one of the last three sexual partners in the last 12 months | Not included. | Not included. |
| Early sexual debut | Defined as reporting an age <= 15 years in response to the question “H*ow old were you when you had vaginal sex for the very first time*?” | Defined as reporting an age <= 15 years in response to the question “H*ow old were you when you had sexual intercourse for the very first time*?” | Defined as reporting an age <= 15 years in response to the question “H*ow old were you when you had sexual intercourse for the very first time*?” |
| Transactional sex (past 12 months)^a^ | Defined as reporting ‘yes’ in response to the questions “*Did you enter into a sexual relationship with [partner] because [partner] provided you (or you expected that [partner] would provide you) with gifts, help you to pay for things, or help in other ways*” for at least one of the last three sexual partners in the last 12 months | Defined as reporting ‘yes’ in response to the question “*In the past 12 months, have you had sex or been sexually involved with anyone because he gave you or told you he would give you gifts, cash, or anything else*?” | Not included. |
| Multiple sex partners (past 12 months) | Defined as reporting more than 1 in response to the question “*With how many different people have you had sex in the last 12 months?”* | Defined as reporting more than 1 sex partner, including spouse, in last 12 months | Defined as reporting more than 1 sex partner, including spouse, in last 12 months |
| Age disparate sex (past 12 months)^b^ | Defined as having an age difference of >= 10 years for each of up to the last three sexual partners in the last 12 months | Defined as having an age difference of >= 10 years for each of up to the last three sexual partners in the last 12 months | Defined as having an age difference of >= 10 years for each of up to the last three sexual partners in the last 12 months |
| Ever forced sex | For AGYW 10-14 (Adolescent Survey):   - Sexual violence was defined as responding ‘yes’ to the question “*Has anyone ever physically forced you to have sex and did succeed*?” | For AGYW 15+ selected for the Domestic Violence module:  Defined as reporting ‘yes’ in response to any of the following questions:   - (Does/did) your (last) husband/partner ever:   - Physically force you to have sexual intercourse with him even when you did not want to?   - Force you to perform other sexual acts you did not want to?   - Force you to perform other sexual acts you did not want to? - First intercourse was unwanted or forced - Ever forced to have sex by anyone other than husband/partner in last 12 months - Ever forced to perform unwanted sexual acts - Previous husband/partner physically force you to have intercourse or perform any other sexual acts against your will? | For AGYW 15+ selected for the Domestic Violence module:  Defined as reporting ‘yes’ in response to any of the following questions:   - (Does/did) your (last) husband/partner ever:   - Physically force you to have sexual intercourse with him even when you did not want to?   - Force you to perform other sexual acts you did not want to?   - Force you to perform other sexual acts you did not want to? - First intercourse was unwanted or forced - Ever forced to have sex by anyone other than husband/partner in last 12 months - Ever forced to perform unwanted sexual acts - Previous husband/partner physically forced you to have intercourse or perform any other sexual acts against your will? - Ever been physically forced into unwanted sex by (last) husband/partner - Ever been physically forced to perform sexual acts respondent didn't want to by (last) husband/partner - Ever been forced into other unwanted sexual acts by (last) husband/partner |
| Ever experienced physical violence | For AGYW 10-14 (Adolescent Survey):   - Sexual violence was defined as responding ‘yes’ to the question “*Has anyone ever physically forced you to have sex and did succeed*?” | For AGYW 15+ selected for the Domestic Violence module:  Defined as reporting ‘yes’ in response to any of the following questions:   - (Does/did) your (last) husband/partner ever:   - Push, shake, or throw something at you; slap you or twist your arm, punch you with his fist or with something that could hurt you   - Kick you or drag you; try to strangle you or burn you; threaten you with a knife, gun, or other type of weapon   - Physically force you to have sexual intercourse or sexual acts when you did not want to; force you to perform other unwanted sexual acts - Has anyone (other than your husband/partner) hit you, slapped you, or done anything else to hurt you physically? - Has anyone ever hit, slapped, kicked, or done anything else to hurt you physically while you were pregnant? - Did your previous husband/partner ever hit, slap, kick or physically hurt you? | For AGYW 15+ selected for the Domestic Violence module:  Defined as reporting ‘yes’ in response to any of the following questions:   - (Does/did) your (last) husband/partner ever:   - Push, shake, or throw something at you; pull your hair or twist your arm, punch you with his fist or with something that could hurt you   - Kick you or drag you; try to strangle you or burn you; threaten you with a knife, gun, or other type of weapon   - Physically force you to have sexual intercourse or sexual acts when you did not want to; force you to perform other unwanted sexual acts - Has anyone (other than your husband/partner) hit you, slapped you, or done anything else to hurt you physically? - Has anyone ever hit, slapped, kicked, or done anything else to hurt you physically while you were pregnant? - Did your previous husband/partner ever hit, slap, kick or physically hurt you? |
| Ever experienced violence (physical or sexual or emotional)^d^ | For AGYW 10-14 (Adolescent Survey),  Coded as responding yes to either ever experienced physical violence or ever experienced sexual violence | For AGYW 15+, coded as responding yes to either ever experienced physical violence or ever experienced sexual violence or emotional violence (defined as ever being humiliated, threatened by harm, or insulted/made to feel bad by husband/partner) | For AGYW 15+, coded as responding yes to either ever experienced physical violence or ever experienced sexual violence or emotional violence (defined as ever being humiliated, threatened by harm, or insulted/made to feel bad by husband/partner) |
| Current experience of violence in past 12 months (physical and/or sexual)^d^ | For AGYW 15+:   - Sexual violence was defined as responding ‘yes’ to the question “*In the past 12 months, did someone physically force you to have sex*?” - Physical violence was defined as reporting ‘once’, ‘few’, or ‘many’ in response to the question “*In the past 12 months, how many times did someone punched, kicked, whipped, or beat you with an object; slapped you, threw something at you that could hurt you, pushed you or shoved you; choked, smothered, tried to drown you or burned you intentionally, or used or threatened you with a knife, gun, or other weapon?”* | Not included. | Not included. |
| Current experience of violence in past month (physical discipline) | Not included. | Note that the Child Discipline module is administered to a knowledgeable adult, who responds to questions about one child in randomly selected households.  Defined as an adult reporting ‘yes’ in response to the question: “*Have you or anyone else in the household used this method with (NAME) in the past month: shook him/her; pull his/her ears; hit him/her on the bottom with bare hand; hit him/her on the bottom or elsewhere on the body with a belt, hairbrush, stick or other; hit or slapped him/her on the face, head or ears; hit or slapped him/her on the hand, arm or leg; beat him/her up with an implement*” | Not included. |
| Exposure to childhood physical abuse in household (past month) | Not included. | Defined as a child living in a household in which any child has experienced physical violent discipline in the past month | Not included. |
| Consumes alcohol regularly | Defined as responding “2-3 times a week” or “4 or more times a week” to the question “*How often do you have a drink containing alcohol*?” | Defined as drinking alcohol every day, or every now and then | Not included. |
| Alcohol use^e^ | Not included. | Not included. | Not included. |
| Uses tobacco | Not included. | Defined as currently smoking cigarettes every day/some days, or currently smoking or using pipes full of tobacco, chewing tobacco, snuff by nose, or other | Not included. |
| Single orphan | Defined as an adult responding “no” to one of the following questions: “*Is [child’s] natural mother alive*? Or “*Is [child’s] natural father alive*?” for children in household roster module | Defined as an adult responding “no” to one of the following questions: “*Is [child’s] natural mother alive*? Or “*Is [child’s] natural father alive*?” for children in household roster module | Defined as an adult responding “no” to one of the following questions: “*Is [child’s] natural mother alive*? Or “*Is [child’s] natural father alive*?” for children in household roster module |
| Double orphan | Defined as an adult responding “no” to both of the following questions: “*Is [child’s] natural mother alive*? Or “*Is [child’s] natural father alive*?” for children in household roster module | Defined as an adult responding “no” to both of the following questions: “*Is [child’s] natural mother alive*? Or “*Is [child’s] natural father alive*?” for children in household roster module | Defined as an adult responding “no” to both of the following questions: “*Is [child’s] natural mother alive*? Or “*Is [child’s] natural father alive*?” for children in household roster module |
| Orphanhood (either single or double) | Defined as responding yes to either the single and double orphan variables | Not included. | Not included. |
| Not currently enrolled in school | Defined as an adult responding “no, currently not in school” or “no, too young to be in school” for the question “*Is [child] enrolled in school*?” for children in household roster module  or  A young adolescent reporting ‘no’ for the question “*Are you enrolled in school*?” | Defined as an adult responding “yes” to the question: “*Did [name] attend school at any time during the current school year?”* for children under 18 in household roster module | Not included. |
| Never attended or not currently enrolled in school | Not included. | Not included. | Defined as adult responding “yes” to the question: “*Did [name] attend school at any time during the current school year?”* or stating no educational attainment for children under 18 in household roster module |
