# Supplementary material for: Characterizing and mapping the spatial variability of HIV risk among adolescent girls and young women: A cross-county analysis of population-based surveys in Eswatini, Haiti, and Mozambique
Source: PLoS One. 2021 Dec 17;16(12):e0261520. doi: 10.1371/journal.pone.0261520 (PMC8682891; doi:10.1371/journal.pone.0261520)
Supplement: S2 Table — (DOCX) [file pone.0261520.s005.docx]

S2 Table. Descriptive statistics by HIV status, by country and age band

Descriptive statistics are presented for AGYW 15-24 in Haiti and Mozambique and AGYW 10-29 in Eswatini with conclusive HIV test results, non-zero biomarker survey weights, and at least one non-missing risk factor variable; sample characteristics are presented for AGYW ages 10-14 years with at least one non-missing risk factor in Haiti and Mozambique. Unweighted numerator (n) and denominator (N) counts and survey weighted, cluster-adjusted percentages and Chi-square tests of difference in characteristic distribution by HIV status are presented.

**Haiti, AGYW ages 10-14**

|  | **Total** | | |
| --- | --- | --- | --- |
| **Risk and vulnerability factor** | **n** | **N** | **%** |
| Self-reported STI status (past 12 months) |  |  |  |
| Sexually active (past 4 weeks) |  |  |  |
| Inconsistent condom use (past 12 months) |  |  |  |
| Early sexual debut at age 15 or younger |  |  |  |
| Transactional sex (past 12 months) |  |  |  |
| Multiple sex partners (past 12 months) |  |  |  |
| Age disparate sex (past 12 months) |  |  |  |
| Ever forced sex |  |  |  |
| Ever experienced physical violence |  |  |  |
| Ever experienced violence (physical, sexual or emotional) |  |  |  |
| Child lives in a household where at least one child has been physically abused (past 12 months) | 1592 | 2139 | 74.69 |
| Child has personally experienced physical violent discipline (past 12 months) | 677 | 1004 | 68.44 |
| Consumes alcohol regularly |  |  |  |
| Uses tobacco |  |  |  |
| Single orphan | 418 | 3244 | 13.22 |
| Double orphan | 47 | 3244 | 1.45 |
| Not currently enrolled in school | 190 | 3249 | 5.49 |

**Haiti, AGYW ages 15-19**

|  | **Total** | | | **HIV-positive**  *(N = 8)* | | | **HIV-negative**  *(N=2223)* | | |  |
| --- | --- | --- | --- | --- | --- | --- | --- | --- | --- | --- |
| **Risk and vulnerability factor** | **n** | **N** | **%** | **n** | **N** | **%** | **n** | **N** | **%** | **p-value** |
| Self-reported STI status (past 12 months) | 89 | 2231 | 4.09 | 0 | 8 | 0 | 89 | 2223 | 4.10 | 0.606‡ |
| Sexually active (past 4 weeks) | 376 | 2231 | 16.77 | 2 | 8 | 21.72 | 374 | 2223 | 16.75 | 0.706‡ |
| Inconsistent condom use (past 12 months) | 153 | 1678 | 8.67 | 2 | 5 | 54.60 | 151 | 1673 | 8.52 | 0.001‡ |
| Early sexual debut at age 15 or younger | 514 | 2231 | 22.33 | 2 | 8 | 13.62 | 512 | 2223 | 22.36 | 0.477‡ |
| Transactional sex (past 12 months) | 26 | 1907 | 1.62 | 0 | 5 | 0 | 26 | 1902 | 1.62 | 0.805‡ |
| Multiple sex partners (past 12 months) | 48 | 2231 | 2.46 | 1 | 8 | 26.73 | 47 | 2223 | 2.38 | 0.259‡ |
| Age disparate sex (past 12 months) | 70 | 2092 | 3.07 | 1 | 8 | 4.91 | 69 | 2084 | 3.06 | 0.649‡ |
| Ever forced sex | 92 | 1122 | 8.17 | 1 | 5 | 17.86 | 91 | 1117 | 8.14 | 0.415‡ |
| Ever experienced physical violence | 309 | 1123 | 29.03 | 3 | 5 | 62.58 | 306 | 1118 | 28.91 | 0.118‡ |
| Ever experienced violence (physical, sexual or emotional) | 349 | 1123 | 32.39 | 4 | 5 | 89.91 | 345 | 1118 | 32.19 | 0.000‡ |
| Child lives in a household where at least one child has been physically abused (past 12 months) | -- | -- | -- | -- | -- | -- | -- | -- | -- | -- |
| Child has personally experienced physical violent discipline (past 12 months) | -- | -- | -- | -- | -- | -- | -- | -- | -- | -- |
| Consumes alcohol regularly | 107 | 2231 | 5.56 | 1 | 8 | 13.02 | 106 | 2223 | 5.54 | 0.378‡ |
| Uses tobacco | 33 | 2231 | 1.69 | 0 | 8 | 0 | 33 | 2223 | 1.70 | 0.751‡ |
| Single orphan | 260 | 1392 | 19.61 | 1 | 3 | 32.43 | 259 | 1389 | 19.59 | 0.576‡ |
| Double orphan | 36 | 1392 | 2.66 | 2 | 3 | 67.57 | 34 | 1389 | 2.54 | 0.000‡ |
| Not currently enrolled in school | 151 | 1408 | 11.23 | 0 | 3 | 0 | 151 | 1405 | 11.25 | 0.545‡ |

‡Chi-square test statistic should be interpreted with caution as at least one cell has fewer than five observations

**Haiti, AGYW ages 20-24**

|  | **Total** | | | **HIV-positive**  *(N=34)* | | | | | **HIV-negative**  *(N=1778)* | | |  |
| --- | --- | --- | --- | --- | --- | --- | --- | --- | --- | --- | --- | --- |
| **Risk and vulnerability factor** | **n** | **N** | **%** | **n** | | **N** | | **%** | **n** | **N** | **%** | **p-value** |
| Self-reported STI status (past 12 months) | 215 | 1812 | 12.23 | 6 | 34 | | 20.66 | | 209 | 1778 | 12.06 | 0.179‡ |
| Sexually active (past 4 weeks) | 760 | 1812 | 43.25 | 15 | 34 | | 51.33 | | 745 | 1778 | 43.08 | 0.386 |
| Inconsistent condom use (past 12 months) | 188 | 717 | 25.64 | 3 | 6 | | 39.52 | | 185 | 711 | 25.54 | 0.478‡ |
| Early sexual debut at age 15 or younger | 471 | 1812 | 27.55 | 17 | 34 | | 59.92 | | 454 | 1778 | 26.90 | 0.000 |
| Transactional sex (past 12 months) | 18 | 971 | 1.91 | 0 | 12 | | 0 | | 18 | 959 | 1.93 | 0.686‡ |
| Multiple sex partners (past 12 months) | 46 | 1812 | 3.54 | 0 | 34 | | 0 | | 46 | 1778 | 3.61 | 0.356‡ |
| Age disparate sex (past 12 months) | 219 | 1652 | 13.64 | 7 | 31 | | 20.65 | | 212 | 1621 | 13.50 | 0.291 |
| Ever forced sex | 113 | 1103 | 10.43 | 3 | 27 | | 15.16 | | 110 | 1076 | 10.30 | 0.291‡ |
| Ever experienced physical violence | 339 | 1103 | 31.31 | 14 | 27 | | 54.19 | | 325 | 1076 | 30.67 | 0.019 |
| Ever experienced violence (physical, sexual or emotional) | 390 | 1103 | 36.34 | 14 | 27 | | 54.19 | | 376 | 1076 | 35.84 | 0.078 |
| Child lives in a household where at least one child has been physically abused (past 12 months) | -- | -- | -- | -- | -- | | -- | | -- | -- | -- | -- |
| Child has personally experienced physical violent discipline (past 12 months) | -- | -- | -- | -- | -- | | -- | | -- | -- | -- | -- |
| Consumes alcohol regularly | 135 | 1812 | 8.24 | 5 | 34 | | 15.25 | | 130 | 1778 | 8.10 | 0.190 |
| Uses tobacco | 37 | 1812 | 2.56 | 1 | 34 | | 4.54 | | 36 | 1778 | 2.52 | 0.559‡ |
| Single orphan |  |  |  |  |  | |  | |  |  |  |  |
| Double orphan |  |  |  |  |  | |  | |  |  |  |  |
| Not currently enrolled in school |  |  |  |  |  | |  | |  |  |  |  |

‡Chi-square test statistic should be interpreted with caution as at least one cell has fewer than five observations

**Mozambique, AGYW ages 10-14**

|  | **Total** | | |
| --- | --- | --- | --- |
| **Risk and vulnerability factor** | **n** | **N** | **%** |
| Self-reported STI status (past 12 months) |  |  |  |
| Sexually active (past 4 weeks) |  |  |  |
| Inconsistent condom use (past 12 months) |  |  |  |
| Early sexual debut at age 15 or younger |  |  |  |
| Multiple sex partners (past 12 months) |  |  |  |
| Age disparate sex (past 12 months) |  |  |  |
| Ever forced sex |  |  |  |
| Ever experienced physical violence |  |  |  |
| Ever experienced violence (physical, sexual or emotional) |  |  |  |
| Single orphan | 379 | 2510 | 13.68 |
| Double orphan | 80 | 2514 | 3.03 |
| Never attended or not currently enrolled in school | 427 | 2518 | 19.73 |

**Mozambique, AGYW ages 15-19**

|  | **Total** | | | **HIV-positive**  (*N=88)* | | | **HIV-negative**  *(N=1283)* | | |  |
| --- | --- | --- | --- | --- | --- | --- | --- | --- | --- | --- |
| **Risk and vulnerability factor** | **n** | **N** | **%** | **n** | **N** | **%** | **n** | **N** | **%** | **p-value** |
| Self-reported STI status (past 12 months) | 37 | 1365 | 2.36 | 4 | 88 | 2.67 | 33 | 1277 | 2.34 | 0.813‡ |
| Sexually active (past 4 weeks) | 485 | 1370 | 38.64 | 37 | 88 | 40.32 | 448 | 1282 | 38.52 | 0.776 |
| Inconsistent condom use (past 12 months) | 87 | 662 | 12.31 | 6 | 32 | 18.20 | 81 | 630 | 11.99 | 0.324 |
| Early sexual debut at age 15 or younger | 454 | 1371 | 34.46 | 38 | 88 | 45.03 | 416 | 1283 | 33.71 | 0.093 |
| Multiple sex partners (past 12 months) | 35 | 1370 | 2.62 | 4 | 88 | 5.31 | 31 | 1282 | 2.43 | 0.143‡ |
| Age disparate sex (past 12 months) | 89 | 1200 | 7.64 | 13 | 74 | 18.87 | 76 | 1126 | 6.90 | 0.001 |
| Ever forced sex | 17 | 276 | 4.54 | 4 | 24 | 14.10 | 13 | 252 | 3.73 | 0.028‡ |
| Ever experienced physical violence | 47 | 277 | 14.76 | 6 | 24 | 23.77 | 41 | 253 | 13.99 | 0.249 |
| Ever experienced violence (physical, sexual or emotional) | 55 | 277 | 18.60 | 8 | 24 | 32.41 | 47 | 253 | 17.43 | 0.149 |
| Single orphan | 149 | 729 | 18.34 | 8 | 31 | 22.46 | 137 | 698 | 18.14 | 0.561 |
| Double orphan | 29 | 729 | 4.25 | 4 | 31 | 16.30 | 25 | 698 | 3.67 | 0.003‡ |
| Never attended or not currently enrolled in school | 775 | 1369 | 62.72 | 59 | 88 | 69.92 | 716 | 1281 | 62.21 | 0.218 |

‡Chi-square test statistic should be interpreted with caution as at least one cell has fewer than five observations

**Mozambique, AGYW ages 20-24**

|  | **Total** | | | **HIV-positive**  *(N=185)* | | | **HIV-negative**  *(N=1037)* | | |  |
| --- | --- | --- | --- | --- | --- | --- | --- | --- | --- | --- |
| **Risk and vulnerability factor** | **n** | **N** | **%** | **n** | **N** | **%** | **n** | **N** | **%** | **p-value** |
| Self-reported STI status (past 12 months) | 74 | 1214 | 5.13 | 17 | 184 | 8.01 | 57 | 1030 | 4.70 | 0.082 |
| Sexually active (past 4 weeks) | 686 | 1221 | 56.73 | 96 | 185 | 49.40 | 590 | 1036 | 57.83 | 0.053 |
| Inconsistent condom use (past 12 months) | 112 | 245 | 45.87 | 19 | 46 | 41.08 | 93 | 199 | 46.91 | 0.536 |
| Early sexual debut at age 15 or younger | 354 | 1221 | 31.67 | 69 | 185 | 40.08 | 285 | 1036 | 30.41 | 0.049 |
| Multiple sex partners (past 12 months) | 48 | 1221 | 3.66 | 12 | 185 | 7.68 | 36 | 1036 | 3.06 | 0.007 |
| Age disparate sex (past 12 months) | 189 | 982 | 20.52 | 42 | 146 | 31.49 | 147 | 836 | 18.93 | 0.002 |
| Ever forced sex | 35 | 612 | 4.88 | 8 | 87 | 8.50 | 27 | 525 | 4.35 | 0.090 |
| Ever experienced physical violence | 147 | 613 | 21.18 | 29 | 87 | 33.31 | 118 | 526 | 19.42 | 0.004 |
| Ever experienced violence (physical, sexual or emotional) | 172 | 613 | 25.00 | 34 | 87 | 37.70 | 138 | 526 | 23.17 | 0.005 |
| Single orphan | -- | -- | -- | -- | -- | -- | -- | -- | -- | -- |
| Double orphan | -- | -- | -- | -- | -- | -- | -- | -- | -- | -- |
| Never attended or not currently enrolled in school | 1041 | 1221 | 88.31 | 165 | 184 | 90.83 | 876 | 1037 | 87.94 | 0.306 |

‡Chi-square test statistic should be interpreted with caution as at least one cell has fewer than five observations

**Eswatini, AGYW ages 10-14**

|  | **Total** | | | **HIV-positive**  (*N=20)* | | | **HIV-negative**  *(N=558)* | | |  |
| --- | --- | --- | --- | --- | --- | --- | --- | --- | --- | --- |
| **Risk and vulnerability factor** | **n** | **N** | **%** | **n** | **N** | **%** | **n** | **N** | **%** | **p-value** |
| Inconsistent condom use (past 12 months) | -- | -- | -- | -- | -- | -- | -- | -- | -- | -- |
| No condom use at last sex | -- | -- | -- | -- | -- | -- | -- | -- | -- | -- |
| Early sexual debut at age 15 or younger | -- | -- | -- | -- | -- | -- | -- | -- | -- | -- |
| Transactional sex (past 12 months) | -- | -- | -- | -- | -- | -- | -- | -- | -- | -- |
| Multiple sex partners (past 12 months) | -- | -- | -- | -- | -- | -- | -- | -- | -- | -- |
| Age disparate sex (past 12 months) | -- | -- | -- | -- | -- | -- | -- | -- | -- | -- |
| Ever forced sex (under 15 only) | 1 | 228 | 0.40 | 0 | 6 | 0 | 1 | 222 | 0.41 | 0.869‡ |
| Ever experienced physical violence (under 15 only) | 43 | 568 | 7.58 | 1 | 20 | 5.51 | 42 | 548 | 7.66 | 0.732‡ |
| Ever experienced violence (physical and/or sexual; under 15 only) | 44 | 569 | 7.72 | 1 | 20 | 5.51 | 43 | 549 | 7.80 | 0.717‡ |
| Experienced forced sex in past 12 months (15+ only) | -- | -- | -- | -- | -- | -- | -- | -- | -- | -- |
| Experienced physical violence in past 12 months (15+ only) | -- | -- | -- | -- | -- | -- | -- | -- | -- | -- |
| Experienced violence in past 12 months (physical and/or sexual; 15+ only) | -- | -- | -- | -- | -- | -- | -- | -- | -- | -- |
| Alcohol use | -- | -- | -- | -- | -- | -- | -- | -- | -- | -- |
| Single orphan | 101 | 556 | 17.79 | 9 | 20 | 44.28 | 92 | 536 | 16.81 | 0.004 |
| Double orphan | 14 | 573 | 2.18 | 1 | 20 | 7.34 | 13 | 553 | 2.00 | 0.186‡ |
| Not currently enrolled in school (under 18 only) | 3 | 578 | 0.66 | 0 | 20 | 0 | 3 | 558 | 0.69 | 0.755‡ |

‡Chi-square test statistic should be interpreted with caution as at least one cell has fewer than five observations

**Eswatini, AGYW ages 15-19**

|  | **Total** | | | **HIV-positive**  (*N=72)* | | | **HIV-negative**  *(N=959)* | | |  |
| --- | --- | --- | --- | --- | --- | --- | --- | --- | --- | --- |
| **Risk and vulnerability factor** | **n** | **N** | **%** | **n** | **N** | **%** | **n** | **N** | **%** | **p-value** |
| Inconsistent condom use (past 12 months) | 164 | 996 | 16.36 | 23 | 71 | 31.34 | 141 | 925 | 15.17 | 0.002 |
| No condom use at last sex | 82 | 263 | 30.65 | 12 | 37 | 32.15 | 70 | 226 | 30.41 | 0.836 |
| Early sexual debut at age 15 or younger | 66 | 1022 | 6.56 | 12 | 72 | 16.83 | 54 | 950 | 5.76 | 0.001 |
| Transactional sex (past 12 months) | 25 | 995 | 2.35 | 4 | 71 | 5.39 | 21 | 924 | 2.10 | 0.061‡ |
| Multiple sex partners (past 12 months) | 22 | 1025 | 2.26 | 5 | 72 | 6.98 | 17 | 953 | 1.90 | 0.017 |
| Age disparate sex (past 12 months) | 34 | 994 | 3.18 | 3 | 70 | 4.15 | 31 | 924 | 3.11 | 0.633‡ |
| Ever forced sex (under 15 only) | -- | -- | -- | -- | -- | -- | -- | -- | -- | -- |
| Ever experienced physical violence (under 15 only) | -- | -- | -- | -- | -- | -- | -- | -- | -- | -- |
| Ever experienced violence (physical and/or sexual; under 15 only) | -- | -- | -- | -- | -- | -- | -- | -- | -- | -- |
| Experienced forced sex in past 12 months (15+ only) | 0 | 291 | 0 | 0 | 22 | 0 | 0 | 269 | 0 |  |
| Experienced physical violence in past 12 months (15+ only) | 2 | 213 | 0.95 | 0 | 19 | 0 | 2 | 194 | 1.05 | 0.667‡ |
| Experienced violence in past 12 months (physical and/or sexual; 15+ only) | 2 | 324 | 0.62 | 0 | 25 | 0 | 2 | 299 | 0.67 | 0.696‡ |
| Alcohol use | 0 | 1024 | 0 | 0 | 71 | 0 | 0 | 953 | 0 |  |
| Single orphan | 174 | 602 | 28.49 | 18 | 32 | 55.46 | 156 | 570 | 26.98 | 0.004 |
| Double orphan | 50 | 616 | 8.10 | 6 | 33 | 18.31 | 44 | 583 | 7.52 | 0.043 |
| Not currently enrolled in school (under 18 only) | 51 | 624 | 7.90 | 6 | 34 | 17.72 | 45 | 590 | 7.30 | 0.040 |

‡Chi-square test statistic should be interpreted with caution as at least one cell has fewer than five observations

**Eswatini, AGYW ages 20-24**

|  | **Total** | | | **HIV-positive**  (*N=197)* | | | **HIV-negative**  *(N=698)* | | |  |
| --- | --- | --- | --- | --- | --- | --- | --- | --- | --- | --- |
| **Risk and vulnerability factor** | **n** | **N** | **%** | **n** | **N** | **%** | **n** | **N** | **%** | **p-value** |
| Inconsistent condom use (past 12 months) | 467 | 823 | 57.33 | 89 | 176 | 51.25 | 378 | 647 | 58.90 | 0.108 |
| No condom use at last sex | 187 | 492 | 37.65 | 41 | 110 | 36.77 | 146 | 382 | 37.88 | 0.844 |
| Early sexual debut at age 15 or younger | 82 | 871 | 9.30 | 31 | 191 | 15.74 | 51 | 680 | 7.59 | 0.003 |
| Transactional sex (past 12 months) | 26 | 824 | 2.99 | 9 | 175 | 5.12 | 17 | 649 | 2.44 | 0.050 |
| Multiple sex partners (past 12 months) | 75 | 879 | 9.01 | 21 | 190 | 11.04 | 54 | 689 | 8.48 | 0.397 |
| Age disparate sex (past 12 months) | 126 | 817 | 15.87 | 46 | 174 | 26.34 | 80 | 643 | 13.19 | 0.001 |
| Ever forced sex (under 15 only) | -- | -- | -- | -- | -- | -- | -- | -- | -- | -- |
| Ever experienced physical violence (under 15 only) | -- | -- | -- | -- | -- | -- | -- | -- | -- | -- |
| Ever experienced violence (physical and/or sexual; under 15 only) | -- | -- | -- | -- | -- | -- | -- | -- | -- | -- |
| Experienced forced sex in past 12 months (15+ only) | 0 | 379 | 0 | 0 | 87 | 0 | 0 | 292 | 0 |  |
| Experienced physical violence in past 12 months (15+ only) | 19 | 283 | 6.31 | 6 | 65 | 9.03 | 13 | 218 | 5.57 | 0.329 |
| Experienced violence in past 12 months (physical and/or sexual; 15+ only) | 19 | 407 | 4.44 | 6 | 94 | 6.29 | 13 | 313 | 3.93 | 0.338 |
| Alcohol use | 5 | 893 | 0.62 | 3 | 197 | 1.98 | 2 | 696 | 0.25 | 0.016‡ |
| Single orphan | -- | -- | -- | -- | -- | -- | -- | -- | -- | -- |
| Double orphan | -- | -- | -- | -- | -- | -- | -- | -- | -- | -- |
| Not currently enrolled in school (under 18 only) | -- | -- | -- | -- | -- | -- | -- | -- | -- | -- |

‡Chi-square test statistic should be interpreted with caution as at least one cell has fewer than five observations

**Eswatini, AGYW ages 25-29**

|  | **Total** | | | **HIV-positive**  (*N=319)* | | | **HIV-negative**  *(N=492)* | | |  |
| --- | --- | --- | --- | --- | --- | --- | --- | --- | --- | --- |
| **Risk and vulnerability factor** | **n** | **N** | **%** | **n** | **N** | **%** | **n** | **N** | **%** | **p-value** |
| Inconsistent condom use (past 12 months) | 488 | 766 | 63.66 | 155 | 297 | 51.60 | 333 | 469 | 70.77 | 0.000 |
| No condom use at last sex | 131 | 346 | 36.08 | 40 | 137 | 29.19 | 91 | 209 | 40.26 | 0.092 |
| Early sexual debut at age 15 or younger | 85 | 788 | 10.02 | 48 | 305 | 15.01 | 37 | 483 | 7.11 | 0.002 |
| Transactional sex (past 12 months) | 21 | 767 | 2.59 | 11 | 297 | 3.92 | 10 | 470 | 1.81 | 0.081 |
| Multiple sex partners (past 12 months) | 57 | 803 | 7.49 | 26 | 315 | 8.67 | 31 | 488 | 6.79 | 0.395 |
| Age disparate sex (past 12 months) | 139 | 765 | 18.09 | 65 | 296 | 21.00 | 74 | 469 | 16.38 | 0.122 |
| Ever forced sex (under 15 only) | -- | -- | -- | -- | -- | -- | -- | -- | -- | -- |
| Ever experienced physical violence (under 15 only) | -- | -- | -- | -- | -- | -- | -- | -- | -- | -- |
| Ever experienced violence (physical and/or sexual; under 15 only) | -- | -- | -- | -- | -- | -- | -- | -- | -- | -- |
| Experienced forced sex in past 12 months (15+ only) | 0 | 434 | 0 | 0 | 177 | 0 | 0 | 257 | 0 |  |
| Experienced physical violence in past 12 months (15+ only) | 19 | 304 | 5.43 | 14 | 128 | 9.93 | 5 | 176 | 2.38 | 0.004 |
| Experienced violence in past 12 months (physical and/or sexual; 15+ only) | 19 | 463 | 3.60 | 14 | 192 | 6.66 | 5 | 271 | 1.57 | 0.003 |
| Alcohol use | 9 | 809 | 0.96 | 5 | 319 | 1.31 | 4 | 490 | 0.75 | 0.405 |
| Single orphan | -- | -- | -- | -- | -- | -- | -- | -- | -- | -- |
| Double orphan | -- | -- | -- | -- | -- | -- | -- | -- | -- | -- |
| Not currently enrolled in school (under 18 only) | -- | -- | -- | -- | -- | -- | -- | -- | -- | -- |

‡Chi-square test statistic should be interpreted with caution as at least one cell has fewer than five observations
