# Supplementary material for: Characterizing and mapping the spatial variability of HIV risk among adolescent girls and young women: A cross-county analysis of population-based surveys in Eswatini, Haiti, and Mozambique
Source: PLoS One. 2021 Dec 17;16(12):e0261520. doi: 10.1371/journal.pone.0261520 (PMC8682891; doi:10.1371/journal.pone.0261520)
Supplement: S3 Table — (DOCX) [file pone.0261520.s006.docx]

**S3 Table. Unweighted contingency tables**

|  | | | **Haiti** | | | **Mozambique** | | | **Eswatini** | | |
| --- | --- | --- | --- | --- | --- | --- | --- | --- | --- | --- | --- |
|  |  |  | **HIV Status** | |  | **HIV Status** | |  | **HIV Status** | |  |
|  | **Age band** | **Risk Status** | **Positive** | **Negative** | **Total** | **Positive** | **Negative** | **Total** | **Positive** | **Negative** | **Total** |
| *Any*  *Risk*  *Profile* | *10-14* | At-risk | **--** | **--** | **--** | **--** | **--** | **--** | 11 | 152 | 163 |
|  |  | Not at-risk | **--** | **--** | **--** | **--** | **--** | **--** | 9 | 406 | 415 |
|  |  | **Total** | **--** | **--** | **--** | **--** | **--** | **--** | 20 | 558 | **578** |
|  | *15-19* | At-risk | 8 | 1142 | 1150 | 84 | 992 | 1076 | 50 | 373 | 423 |
|  |  | Not at-risk | 0 | 1081 | 1081 | 4 | 291 | 295 | 22 | 586 | 608 |
|  |  | **Total** | 8 | 2223 | **2231** | 88 | 1283 | **1371** | 72 | 959 | **1031** |
|  | *20-24* | At-risk | 30 | 1203 | 1233 | 183 | 993 | 1176 | 125 | 426 | 551 |
|  |  | Not at-risk | 4 | 575 | 579 | 2 | 44 | 46 | 72 | 272 | 344 |
|  |  | **Total** | 34 | 1778 | **1812** | 185 | 1037 | **1222** | 197 | 698 | **895** |
|  | *25-29* | At-risk | **--** | **--** | **--** | **--** | **--** | **--** | 216 | 374 | 590 |
|  |  | Not at-risk | **--** | **--** | **--** | **--** | **--** | **--** | 103 | 118 | 221 |
|  |  | **Total** | **--** | **--** | **--** | **--** | **--** | **--** | 319 | 492 | **811** |
| *Regress-*  *ion*  *Based Profile* | *15-19* | At-risk | 3 | 490 | 493 | 51 | 600 | 651 | 18 | 88 | 106 |
|  |  | Not at-risk | 5 | 1594 | 1599 | 23 | 524 | 547 | 51 | 826 | 877 |
|  |  | **Total** | 8 | 2084 | **2092** | 74 | 1124 | **1198** | 69 | 914 | **983** |
|  | *20-24* | At-risk | 20 | 572 | 592 | 113 | 494 | 607 | 79 | 158 | 237 |
|  |  | Not at-risk | 11 | 1049 | 1060 | 31 | 337 | 368 | 92 | 472 | 564 |
|  |  | **Total** | 31 | 1621 | **1652** | 144 | 831 | **975** | 171 | 630 | **801** |
|  | *25-29* | At-risk | -- | **--** | **--** | **--** | **--** | **--** | 122 | 120 | 242 |
|  |  | Not at-risk | **--** | **--** | **--** | **--** | **--** | **--** | 162 | 340 | 502 |
|  |  | **Total** | **--** | **--** | **--** | **--** | **--** | **--** | 284 | 460 | **744** |
| *LCA*  *Based*  *Risk* | *15-19* | At-risk | 6 | 813 | 819 | 68 | 710 | 778 | 17 | 90 | 107 |
|  |  | Not at-risk | 2 | 1410 | 1412 | 20 | 573 | 593 | 55 | 869 | 924 |
|  |  | **Total** | 8 | 2223 | **2231** | 88 | 1233 | **1371** | 72 | 959 | **1031** |
|  | *20-24* | At-risk | 29 | 1099 | 1128 | 171 | 921 | 1092 | 56 | 139 | 195 |
|  |  | Not at-risk | 5 | 679 | 684 | 14 | 116 | 130 | 141 | 559 | 700 |
|  |  | **Total** | 34 | 1778 | **1812** | 185 | 1037 | **1222** | 197 | 698 | **895** |
|  | *25-29* | At-risk | -- | **--** | **--** | **--** | **--** | **--** | 84 | 95 | 179 |
|  |  | Not at-risk | **--** | **--** | **--** | **--** | **--** | **--** | 235 | 397 | 632 |
|  |  | **Total** | **--** | **--** | **--** | **--** | **--** | **--** | 319 | 492 | **811** |
