# Supplementary material for: Characterizing and mapping the spatial variability of HIV risk among adolescent girls and young women: A cross-county analysis of population-based surveys in Eswatini, Haiti, and Mozambique
Source: PLoS One. 2021 Dec 17;16(12):e0261520. doi: 10.1371/journal.pone.0261520 (PMC8682891; doi:10.1371/journal.pone.0261520)
Supplement: S4 Table — (DOCX) [file pone.0261520.s007.docx]

**S4 Table. LCA goodness-of-fit model statistics**

| Country | LCA Risk model | Sample size | Classes | AIC | BIC |
| --- | --- | --- | --- | --- | --- |
| Haiti | Full sample, all risk factors | 4043 | 2 | 30,544 | 30,733 |
|  |  |  | 3 | 29,306 | 29,596 |
|  | Full sample, reduced risk factors | 4043 | 2 | 16,482 | 16,571 |
|  |  |  | 3 | 16,423 | 16,568 |
|  | Complete case sample, reduced risk factors | 3744 | 2 | 15,406 | 15,500 |
|  |  |  | 3 | 15,347 | 15,490 |
| Mozambique | Full sample, all risk factors | 2593 | 2 | 13,693 | 13,793 |
|  |  |  | 3 | 13,644 | 13,790 |
|  | Full sample, reduced risk factors | 2593 | 2 | 11,978 | 12,054 |
|  |  |  | 3 | 11,957 | 12,074 |
|  | Complete case sample, reduced risk factors | 2173 | 2 | 10,128 | 10,202 |
|  |  |  | 3 | 10,094 | 10,202 |
| Eswatini | Full sample, all risk factors | 2737 | 2 | 706,925 | 707,055 |
|  |  |  | 3 | 691,924 | 692,119 |
|  | Full sample, reduced risk factors | 2737 | 2 | 587,464 | 587,541 |
|  |  |  | 3 | 585,622 | 585,741 |
|  | Complete case sample, reduced risk factors | 2528 | 2 | 568,903 | 568,979 |
|  |  |  | 3 | 566,980 | 567,097 |
